# Nestin protects podocyte from injury in lupus nephritis by mitophagy and oxidative stress

**DOI:** 10.1038/s41419-020-2547-4

**Published:** 2020-05-05

**Authors:** Yuexin Tian, Huifang Guo, Xinyan Miao, Jie Xu, Ran Yang, Lu Zhao, Jinxi Liu, Lin Yang, Fan Gao, Wei Zhang, Qingjuan Liu, Shaoguang Sun, Yu Tian, Hongbo Li, Jie Huang, Cunyang Gu, Shuxia Liu, Xiaojuan Feng

**Affiliations:** 1 Department of Pathology, Hebei Key Laboratory of Nephrology, Center of Metabolic Diseases and Cancer Research, Hebei Medical University, 050017 Shijiazhuang, China; 2 Department of Rheumatology, The Second Hospital of Hebei Medical University, 050017 Shijiazhuang, China; 3 Department of Pathology, Hebei Province Hospital of Chinese Medicine, 050017 Shijiazhuang, China; 4 Department of Nephrology, The Second Hospital of Hebei Medical University, 050017 Shijiazhuang, China; 5 Department of Biochemistry and Molecular Biology, Hebei Medical University, 050017 Shijiazhuang, China

**Keywords:** Mitophagy, Intermediate filaments, Mechanisms of disease, Prognostic markers, Lupus nephritis

## Abstract

Podocyte injury is the main cause of proteinuria in lupus nephritis (LN). Nestin, an important cytoskeleton protein, is expressed stably in podocytes and is associated with podocyte injury. However, the role of nestin in the pathogenesis of proteinuria in LN remains unclear. The correlations among nestin, nephrin and proteinuria were analyzed in LN patients and MRL/lpr lupus-prone mice. The expression of nestin in mouse podocyte lines (MPCs) and MRL/lpr mice was knocked down to determine the role of nestin in podocyte injury. Inhibitors and RNAi method were used to explore the role of mitophagy and oxidative stress in nestin protection of podocyte from damage. There was a significantly negative correlation between nestin and proteinuria both in LN patients and MRL/lpr mice, whereas the expression of nephrin was positively correlated with nestin. Knockdown of nestin resulted in not only the decrease of nephrin, p-nephrin (Y1217) and mitophagy-associated proteins in cultured podocytes and the podocytes of MRL/lpr mice, but also mitochondrial dysfunction in podocytes stimulated with LN plasma. The expression and phosphorylation of nephrin was significantly decreased by reducing the level of mitophagy or production of reactive oxygen species (ROS) in cultured podocytes. Our findings suggested that nestin regulated the expression of nephrin through mitophagy and oxidative stress to protect the podocytes from injury in LN.

## Introduction

Long-term severe proteinuria is one of the main clinical manifestations of lupus nephritis (LN), one of the most severe and frequent complication of systemic lupus erythematosus (SLE), and a sign of poor prognosis^1^. The patients with LN have varying degrees of renal injury and proteinuria, and proteinuria levels are also unstable in the LN patients, which are associated with a strong self-renewal and repair system of the glomerular filtration membrane^2^. In the early LN, it is possible to repair the pathological damage to filtration membrane caused by various complements and cytokines through self-regulation. However, as the disease progresses, the injury cannot be completely repaired, thereby leading to severe proteinuria^3^. Therefore, it is of great significance and urgency to explore the self-repair mechanism of glomerular filtration membrane in early-stage LN and its failure mechanism in late-stage LN, thereby further preventing proteinuria and relieving renal damage in LN patients.

In a variety of distinct mechanisms of proteinuria, the podocytes dysfunction or injury is the most important cause^4,5^. Nestin is specifically expressed in differentiated and mature podocytes^6–8^. Knocking out the expression of nestin in mice podocytes could cause extensive disappearance or fusion of the foot processes^9^. In addition, the expression of nestin protein in the glomerular podocytes increased transiently and then decreased followed by increased proteinuria while the wistar rats were injected with aminonucleoside puromycin (PAN)^10^. Our previous experiments have confirmed that nestin protein was elevated in high-glucose-stimulated podocytes and diabetic rats and was associated with proteinuria levels^11^. However, the relationship between the expression of nestin and the podocyte injury or proteinuria in LN is poorly understood.

Podocyte-induced proteinuria is associated with the expression of pore-membrane protein. Nephrin is an important component of the fissure membrane, and its abnormal expression indicates damage to the membrane. Researches have shown that the mutation of the phosphorylation site of nephrin could cause the abruption of podocyte foot processes and severe proteinuria^12^, and phosphorylation and dephosphorylation of nephrin tyrosine residues are mainly regulated by fyn and protein tyrosine phosphatase 1B (PTP1B). Fyn belongs to protein tyrosine kinase (PTKs) family, and PTP1B, a group of reactive oxygen species (ROS)-sensitive enzymes, belongs to the protein tyrosine phosphatase (PTPs) family^13^. However, what is the relationship between nestin and nephrin?, and how the phosphorylation level of nephrin is not thoroughly understood.

In this study, firstly we observed the relationship between nestin and proteinuria in patients and mice with LN. Then we elucidated whether nestin protected podocytes from injury by regulating nephrin expression and phosphorylation, through regulating mitophagy and oxidative stress.

## Materials and methods

### Patients and samples

Thirty-two patients diagnosed as II-V LN (ISN/RPS2003 taxonomy) were recruited from the Inpatient Department of Nephrology, the Second Hospital of Hebei Medical University from 2015 to 2018, including 7 males and 25 females, aged 22−50 years old. Percutaneous puncture was performed under ultrasound guidance for renal biopsy. We divided the LN group to two groups according to the mean proteinuria (3.63 g/24 h): mild proteinuria group (LN-MP) and severe proteinuria group (LN-SP) (LN-MP group UPro < 3.63 g/24 h; LN-SP group UPro > 3.63 g/24 h). The 20 control renal tissues were obtained from patients with renal tumors during operation, such as renal clear cell carcinoma and renal leiomyoma, without primary glomerulonephritis, hypertension, diabetic nephropathy or other history of autoimmune diseases, pathologically diagnosed as normal kidney tissue, as age and gender consistent with the LN group. Renal tissues were fixed with 4% formaldehyde for hematoxylin and eosin (HE), periodic acid-Schiff staining (PAS), immunohistochemistry (IHC) and immunofluorescence (IF).

Plasma samples were acquired from five patients with active LN (UPro = 2.848 ± 1.10 g/24 h), without the initiation of immunosuppressive therapy, infections or other complications and underwent therapeutic plasma exchange. All patients met the 2012 revised criteria for SLE and LN and were diagnosed at the Department of Rheumatology of the Second Hospital of Hebei Medical University from August 2016 to June 2017. Five plasma samples were collected from healthy donors, whose age and sex matched the LN patients, and named as the control group. The study was approved by the Clinical Research Ethics Committee of the Second Hospital of Hebei Medical University. Written informed consent was obtained from each study participant.

### Animals

Female MRL/lpr lupus-prone mice and MRL/MPJ mice were purchased from Jackson Laboratory (#JAS000485, Bar Harbor, ME, USA, RRID: IMSR_JAX: 000485). The MRL/lpr mice are susceptible to lupus and are consistent with LN patients in terms of pathological change and clinical performance. All the animal experiments were approved by the Institutional Animal Care and Use Committee of Hebei Medical University (approval ID: HebMU 20080026). The mice weighting 30−35 g were fed in standard environment with regular light/dark cycles and free access to water and food diet. The MRL/lpr lupus-prone mice aged 30 weeks were divided into two groups according to the level of proteinuria: mild proteinuria MRL/lpr mice (MRL/lpr-MP) and severe proteinuria MRL/lpr mice (MRL/lpr-SP) (MRL/lpr-MP group UPro < 43.51 mg/24 h, *n* = 10; MRL/lpr-SP group UPro > 43.51 mg/24 h, *n* = 10). Ten MRL/MPJ mice, whose age and weight matched the MRL/lpr mice, served as the control group. In addition, 18 MRL/lpr mice, aged 27 weeks, were randomly divided into three groups: MRL/lpr group, MRL/lpr-shNC-Ad group, and MRL/lpr-shNestin-Ad group (Cyagen Biotechnology Co., Ltd, Santa Clara, California, USA). The mice in the MRL/lpr-shNC-Ad group and the MRL/lpr-shNestin-Ad group were renally injected with 50 μL 1 × 10^9^ infective units of adenoviruses in both kidneys^14^. Six MRL/lpr mice and six MRL/MPJ mice were injected with isometric saline. After 3 weeks, the mice were sacrificed at age 30-week-old after collecting the 24-h urine and blood samples, and the renal cortex was collected for relevant investigations. The 24 h proteinuria was detected by a mouse urine protein enzyme-linked immunosorbent assay quantitation kit according to the manufacturer’s protocol (ZCi Bio, Shanghai, China, #ZC-38527).

### Immunofluorescence

IF staining was carried out as described in our precious study^15^. The antibody concentration was anti-nestin (1:200; Abcam, Cambridge, MA, USA, #ab11306, RRID: AB_1640723 and #ab22035, RRID: AB_446723), anti-synaptopodin (1:200; Proteintech, Rosemont, IL, USA, #21064-1-AP, RRID: AB_10733120), anti-light chain 3 (LC3, 1:200; Proteintech, #12135-1-AP, RRID: AB_2281381), anti-p62 (1:200; Proteintech, #18420-1-AP, RRID: AB_10694431) and anti-PTEN-induced putative kinase protein 1 (PINK1, 1:200; Proteintech, #23274-1-AP). The images of the section and cells were captured with laser scanning confocal microscope (Leica, Wetzlar, Germany). Image Pro Plus (Media Cybernetics, Silver Spring, MD) was used to quantify the results, and the average integrated optical density (IOD) value was quantified to indicate the expression of protein.

### Immunohistochemistry

The 4% formaldehyde-fixed renal tissue sections were deparaffinized in xylene and rehydrated through graded ethanol. After antigen recovery was performed using a pressure cooker, the endogenous peroxidase was blocked with 3% H_2_O_2_ for 30 min at room temperature. Then the slides were blocked with 10% goat serum and were respectively incubated with primary antibodies against nestin (1:200; Abcam, #ab11306, RRID: AB_1640723 and #ab22035, RRID: AB_446723) and nephrin (1:200; Santa, Dallas, TX, USA, #sc-377246), Mfn1 (1:200; Proteintech, #13798-1-AP, RRID: AB_2266318) and Drp1 (1:200; Abcam, #ab184247) overnight at 4 °C. Then the sections were incubated with polymer helper and polyperoxidase-anti-mouse/rabbit IgG at 37 °C, and finally stained with diaminobenzidine. Finally images were captured using an Olympus microscope (OLYMPUS, BX71, Tokyo, Japan). The average integrated optical density value was quantified to indicate the expression of protein by Image Pro Plus (Media Cybernetics).

### Western blotting

The total protein from MPCs or the renal cortex of mice was extracted by the RIPA lysis buffer. Equal amount of protein extracts (30 μg) was separated by 10 or 12% SDS-PAGE and then transferred to PVDF membranes (Millipore, Billerica, MA, USA). The membranes were blocked with 5% BSA (albumin from bovine serum) for 1.5 h at 37 °C. Then the membranes were incubated overnight at 4 °C with a 1:1000 dilution of nestin (Abcam, #ab11306, RRID: AB_1640723), nephrin (Abcam, #ab58968, RRID: AB_944400), p-nephrin-Y1217 (Abcam, #ab80298, RRID: AB_1603375), PINK1 (Proteintech, 23274-1-AP), LC3 (Proteintech, #12135-1-AP, RRID: AB_2281381), p62 (Proteintech, #18420-1-AP, RRID: AB_10694431), Atg5 (CST, Danvers, MA, USA, #12994, RRID: AB_2630393), Atg12 (CST, #4180, RRID: AB_1903898), Atg16 (CST, #8089, RRID: AB_10950320), Mfn1 (Proteintech, #13798-1-AP, RRID: AB_2266318) and Drp1 (Abcam, #ab184247) antibody. On the second day, the membranes were incubated with a horseradish peroxidase-conjugated goat anti-rabbit or mouse IgG antibody (1:5000; Proteintech, #SA00001-2, RRID: AB_2722564 and #SA00001-1, RRID: AB_2722565). After washing, the images were captured using the LI-COR Odyssey Infrared Imaging System (Lincoln, NE, USA). All experiments were repeated at least three times.

### Cell culture and groups

The immortalized mouse podocyte cell lines (MPCs) were purchased from the Chinese Academy of Sciences, and were cultured in DMEM-F12 (5:1) medium (Thermo Fisher Scientific, Waltham, MA, USA) supplemented with 10% fetal bovine serum (FBS, Thermo Fisher Scientific) at two different temperatures. At 33 °C, cells were allowed to proliferate (permissive condition) in the presence of 10−50 U/mL mouse recombinant IFN-γ (Proteintech, HZ-1301). For the induction of differentiation (nonpermissive condition), podocytes were thermo-shifted to 37 °C in the absence of IFN-γ for 14 days. (1) To investigate the effect of LN plasma on the expression of nestin, nephrin, p-nephrin, Mfn1, Drp1, autophagy-associated proteins, fyn and PTP1B, ROS production and ATP level, the MPCs were randomly exposed to LN plasma (10%) and collected at 0, 12, 24, 36 and 48 h, and Western blot, IF, MitoSOX and flow cytometry (FCM) techniques were used. (2) To further explore the role of nestin in the phosphorylation of nephrin, mitophagy and the generation of ROS in MPCs, the MPCs were randomly divided into 11 groups: control FBS, shNestin, shNC, WT-Nestin, NC, control plasma, LN plasma, LN plasma + shNestin, LN plasma + shNC, LN plasma + WT-Nestin, and LN plasma + NC group, and the cells were collected at 24 h treated with control plasma (10%) or LN plasma (10%). The expression of nestin, nephrin, p-nephrin, Mfn1, Drp1, autophagy-associated proteins, fyn, PTP1B was detected by Western blot; ROS production was measured by the MitoSOX and FCM techniques; and the mitochondrial function and morphology were detected by JC-1 and Mito Tracker, respectively. (3) To explore the role of mitophagy in nestin, expression and phosphorylation of nephrin and the production of ROS, the MPCs were randomly divided into five groups: control plasma, LN plasma, LN plasma + siPINK1, LN plasma + siNC and LN plasma + 3-MA (3-Methyladenine) group. The cells were pretreated with 3-MA (5 mM; MCE, New Jersey, USA, #HY-19312) for 30 min or transfected with siPINK1 and then stimulated with control plasma (10%) or LN plasma (10%). At 24 h, the cells were collected and related indicators were detected. (4) To further investigate the relationship between the ROS production and nestin, mitophagy, nephrin and p-nephrin (Y1217), the MPCs were randomly divided into five groups: control plasma, LN plasma, LN plasma + *N*-acetyl cysteine (NAC), LN plasma + Mito-TEMPO, LN plasma + DMSO group. The MPCs in LN plasma + NAC and LN plasma + Mito-TEMPO were pretreated with NAC (2 mM; Aladdin, Shanghai, China, #A105422) and Mito-TEMPO (10 μM; MCE, HY-112879) for 60 min and then stimulated with LN plasma (10%) for 24 h, and the MPCs in the LN plasma + DMSO group were stimulated with isometric DMSO. And Western blot was used to detect the expression of nestin, mitophagy-associated proteins, PTP1B, nephrin and p-nephrin (Y1217). (5) To investigate whether the PTP1B/Fyn imbalance could cause phosphorylation of nephrin, we randomly divided the MPCs into five groups: control plasma, LN plasma, LN plasma + PTP1B inhibitor, LN plasma + SU6656, and LN plasma + DMSO group. The MPCs in LN plasma + PTP1B inhibitor and LN plasma + SU6656 group were pretreated with PTP1B inhibitor (10 μM; APExBIO, Houston, Texas, USA, #C3772) for 60 min, and SU6656 (10 μM; APExBIO, #B5839) for 30 min and then stimulated by LN plasma (10%) for 24 h. And isometric DMSO was used to stimulate the MPCs in the LN plasma + DMSO group. Then Western blot was used to detect the expression of PTP1B, fyn, nephrin and p-nephrin (Y1217).

### Plasmids and transfection

MPCs were transfected with Nestin-shRNA, Nestin-WT (Cyagen) or siPINK1 (RIBOBIO, Guangzhou, China, #siB160329020753-1-5) using Lipofectamine 3000 according to the manufacturer’s protocols (Invitrogen, Carlsbad, CA, USA, #L3000015). The mRFP-GFP-LC3 plasmid was kindly provided by Prof Liu. The rationale of this assay was based on the pH difference between the acidic autolysosome and the neutral autophagosome, and the pH sensitivity differences exhibited by green fluorescent protein (GFP) and red fluorescent protein (RFP) to monitor progression from autophagosome to autolysosome. When an autophagosome fuses with a lysosome to form an autolysosome, the GFP moiety degrades from the tandem protein, but mRFP-LC3 maintains the puncta, and the fluorescence was observed with a laser scanning confocal microscope (Leica, Wetzlar, Germany). GFP, mRFP and the merged dots/cell were used for quantitation of autophagy flux, and 20 cells were counted in each group.

### Electron microscopy

The renal cortex of the mice was sectioned into 1 mm^3^ cubes with a cold blade and fixed with 2.5% glutaraldehyde. The ultrastructure of the podocytes in renal cortex was observed with a transmission electron microscopy (TEM).

### Mitochondrial membrane potential

The mitochondrial membrane potential (MMP) of MPCs was monitored using JC-1 (Invitrogen, #M34152). Firstly, cells were seeded on slides. After treatment, the cells were incubated with JC-1 for 20 min at 37 °C in the dark. Then the cells were washed with PBS, and the fluorescence was observed with a laser scanning confocal microscope (Leica, Wetzlar, Germany).

### ATP measurement

In accordance with the standard experimental protocol of Beyotime (PRC), cultured cells were homogenized after LN plasma treatment, and the ATP content in MPCs was determined using an ATP Bioluminescence Assay Kit (Beyotime, Shanghai, China, #S0027) according to the manufacturer’s instructions with a luminescence plate reader (Thermo Fisher Scientific) and an integration time of 10 s.

### Mito Tracker staining

After treatment, the above cells were collected and incubated with 250 nM Mito Tracker Red (Invitrogen, #M7512) for 30 min at 37 °C in the dark. Then the cells were washed by PBS, and observed using a laser scanning confocal microscope (Leica, Wetzlar, Germany). Image Pro Plus was used to quantify the results.

### MitoSOX staining

After treatment, the above cells were collected and incubated with 5 μM MitoSOX Red (Invitrogen, #M36008) for 15 min at 37 °C in the dark. Then the cells were washed by HBSS (Hank’s balanced salt solution), and observed using a laser scanning confocal microscope (Leica, Wetzlar, Germany).

### Flow cytometry

The MPCs were treated as described above and incubated with 10 μM DCFH-DA (Beyotime, #S0033) for 20 min at 37 °C. Then the cells were washed with PBS, and the analysis of the fluorescence intensity was performed by a FACSCalibur flow cytometer with CellQuest software (Beckman, Brea, CA, USA).

### PAS staining

After dewaxing and rehydration, 2-μm paraffin sections were stained with PAS. Light microscopy (OLYMPUS, BX71) was used to observe morphological changes in the glomeruli, including cell proliferation, basement membrane thickening, and mesangial areas.

### Statistical analysis

Results were expressed as the mean ± standard error of mean (SEM). SPSS 21.0 (SPSS, Inc., Chicago, IL) was used for data analysis. Student’s *t* test and Mann−Whitney nonparametric tests were applied to compare the variables between the two groups. One-way analysis of variance (ANOVA) was performed to evaluate the statistical significance between multiple comparisons by Bonferroni’s correction. A *P* value < 0.05 was considered statistically significant.

## Results

### Nestin contributed to the proteinuria formation by regulating nephrin in lupus nephritis

**Fig. 1:**
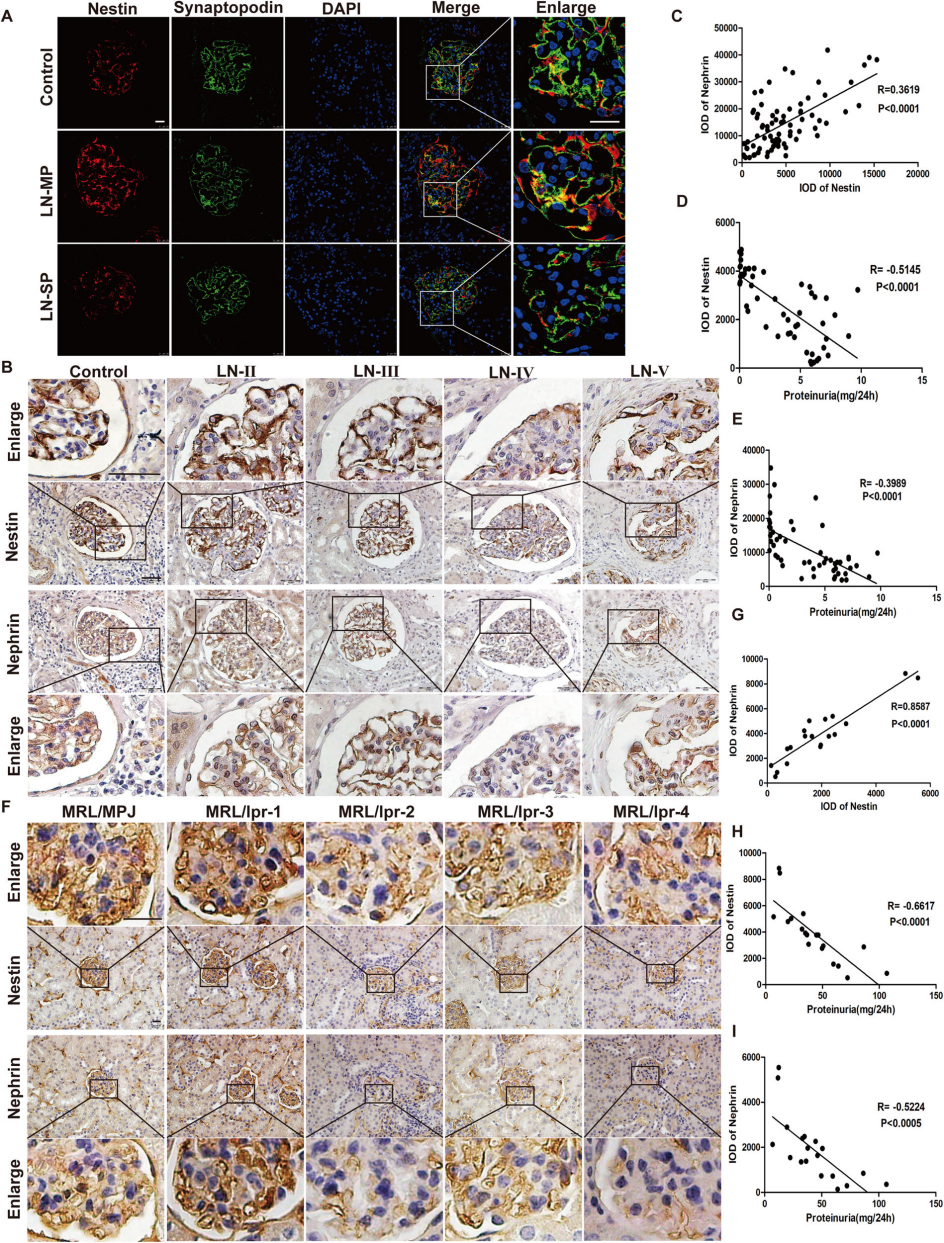
Nestin was consistent with nephrin expression, and negatively correlated with proteinuria in lupus nephritis. **a** Expression of nestin protein and synaptopodin in the glomerulus of LN patients was detected by immunofluorescence. Scale bars: 25 μm. **b** Expression of nestin and nephrin protein in glomerulus of LN patients in different stages was detected by immunohistochemistry. Scale bars: 50 μm. **c** There was a significantly positive correlation between nestin expression and nephrin expression in LN patients (*n* = 80 glomeruli). **d** There was a significantly negative correlation between nestin expression and proteinuria level in LN patients (*n* = 52 patients). **e** There was a significantly negative correlation between nephrin expression and proteinuria level in LN patients (*n* = 52 patients). **f** Expression of nestin and nephrin protein in glomerulus of MRL/lpr mice was detected by immunohistochemistry. Scale bars: 50 μm. **g** There was a significantly positive correlation between nestin and nephrin expression in MRL/lpr mice (*n* = 19 mice). **h** There was a significantly negative correlation between nestin expression and proteinuria level in MRL/lpr mice (*n* = 19 mice). **i** There was a significantly negative correlation between nephrin expression and proteinuria level in MRL/lpr mice (*n* = 19 mice).

Our previous study has proved that abnormal nestin expression played an important role in regulating proteinuria in diabetic nephropathy^11^. To determine the relationship between nestin and proteinuria in LN, the LN patients were divided into two groups according to the proteinuria level. The control renal tissues were obtained from patients with renal tumors during operation, pathologically diagnosed as normal kidney tissues (Supplementary Fig. S1a). As shown in Fig. 1a, the nestin expression was colocated with synaptopodin in podocytes of glomeruli, and nestin expression increased in podocytes in the LN-MP group compared with the control group. Importantly, a notable decrease in nestin was observed in the LN-SP group compared with the LN-MP group, suggesting the potential correlation between nestin and proteinuria.

To further investigate the possible mechanism of nestin in the formation of proteinuria of LN, IHC staining was used to analyze the location and expression of nephrin along with nestin in LN patients. Interestingly, as illustrated in Fig. 1b, c, the nephrin was significantly correlated with nestin in the glomeruli of LN patients. Specifically, nephrin expression was decreased with lower nestin in late LN patients with aggravated proteinuria. Moreover, there was a significantly negative correlation between proteinuria and nestin or nephrin (Fig. 1d, e).

**Fig. 2:**
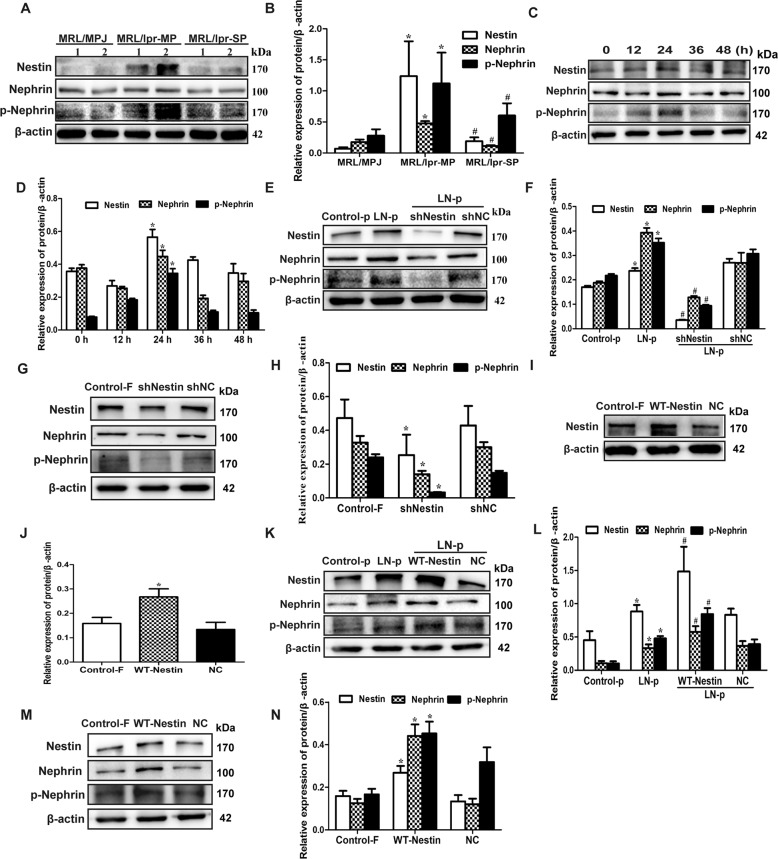
Nestin regulated the expression and phosphorylation of nephrin in MPCs stimulated with LN plasma. **a**, **b** Expression of nestin, nephrin and p-nephrin (Y1217) protein in renal cortex of MRL/lpr mice was detected by Western blot. *P < 0.05 vs. MRL/MPJ mice, ^#^*P* < 0.05 vs. MRL/lpr-MP mice (*n* = 3). **c**, **d** Western blot assay showed the nestin, nephrin and p-nephrin (Y1217) expression in MPCs cultured with 10% LN plasma. *P < 0.05 vs. 0 h group (*n* = 3). **e**, **f** Western blot assay showed the nestin, nephrin and p-nephrin (Y1217) expression in MPCs, which knocked down nestin expression and treated with 10% LN plasma for 24 h. *P < 0.05 vs. control plasma group, ^#^*P* < 0.05 vs. LN plasma + shNC group (*n* = 3). **g**, **h** Western blot assay showed the nestin, nephrin and p-nephrin (Y1217) expression in MPCs, which knocked down nestin expression. *P < 0.05 vs. shNC group (*n* = 3). **i**, **j** Western blot assay showed the nestin expression in MPCs after transfected with the WT-Nestin plasmid. *P < 0.05 vs. NC group (*n* = 3). **k**, **l** Western blot assay showed the nestin, nephrin and p-nephrin (Y1217) expression in MPCs, which was transfected with WT-Nestin plasmid and treated with 10% LN plasma for 24 h. *P < 0.05 vs. control plasma group, ^#^*P* < 0.05 vs. LN plasma + NC group (*n* = 3). **m**, **n** Western blot assay showed the nestin, nephrin and p-nephrin (Y1217) expression in MPCs, which upregulated nestin expression by transfection with WT-Nestin plasmid. *P < 0.05 vs. NC group (*n* = 3). Bonferroni’s correction was performed to analyze statistical significance. Values are mean ± SEM.

Furthermore, we determined the relationship between nestin and nephrin protein in MRL/lpr mice. Similar to LN patients, the results in mice showed that the nephrin was positively correlated with nestin and negatively with proteinuria (Fig. 1f–i). In the meantime, we detected p-nephrin (Y1217) expression. The results showed that nestin, nephrin and p-nephrin (Y1217) expression significantly increased in MRL/lpr-MP mice compared with MRL/MPJ mice, while it significantly decreased in MRL/lpr-SP mice compared with MRL/lpr-MP mice (Fig. 2a, b). These results indicated that nestin and nephrin might be related to the proteinuria that was involved in the pathogenesis of LN.

To clarify the mechanism of differential nephrin expression and whether nestin mediated the injury of podocyte by regulating nephrin, RNAi technique was used in MPCs. Figure 2c, d shows that nestin, nephrin and p-nephrin (Y1217) expression increased in MPCs stimulated by LN plasma at 24 h, and then decreased. Compared with the control FBS group, there was no difference in nestin, nephrin and p-nephrin expression in the control plasma group (Supplementary Fig. S1b, c). Then, as shown in Fig. 2e–h, knockdown of nestin significantly decreased the nephrin and p-nephrin expression in MPCs. After transfection with WT-Nestin plasmid, the protein level of nestin was significantly elevated as compared with the NC group (Fig. 2i, j). However, overexpression of nestin could upregulate the nephrin and p-nephrin (Y1217) expression in MPCs (Fig. 2k–n). The results indicated that nestin might be involved in podocyte injury and proteinuria formation via regulating the expression and phosphorylation of nephrin.

### Nestin regulated the expression and phosphorylation of nephrin by enhancing the mitophagy in LN

**Fig. 3:**
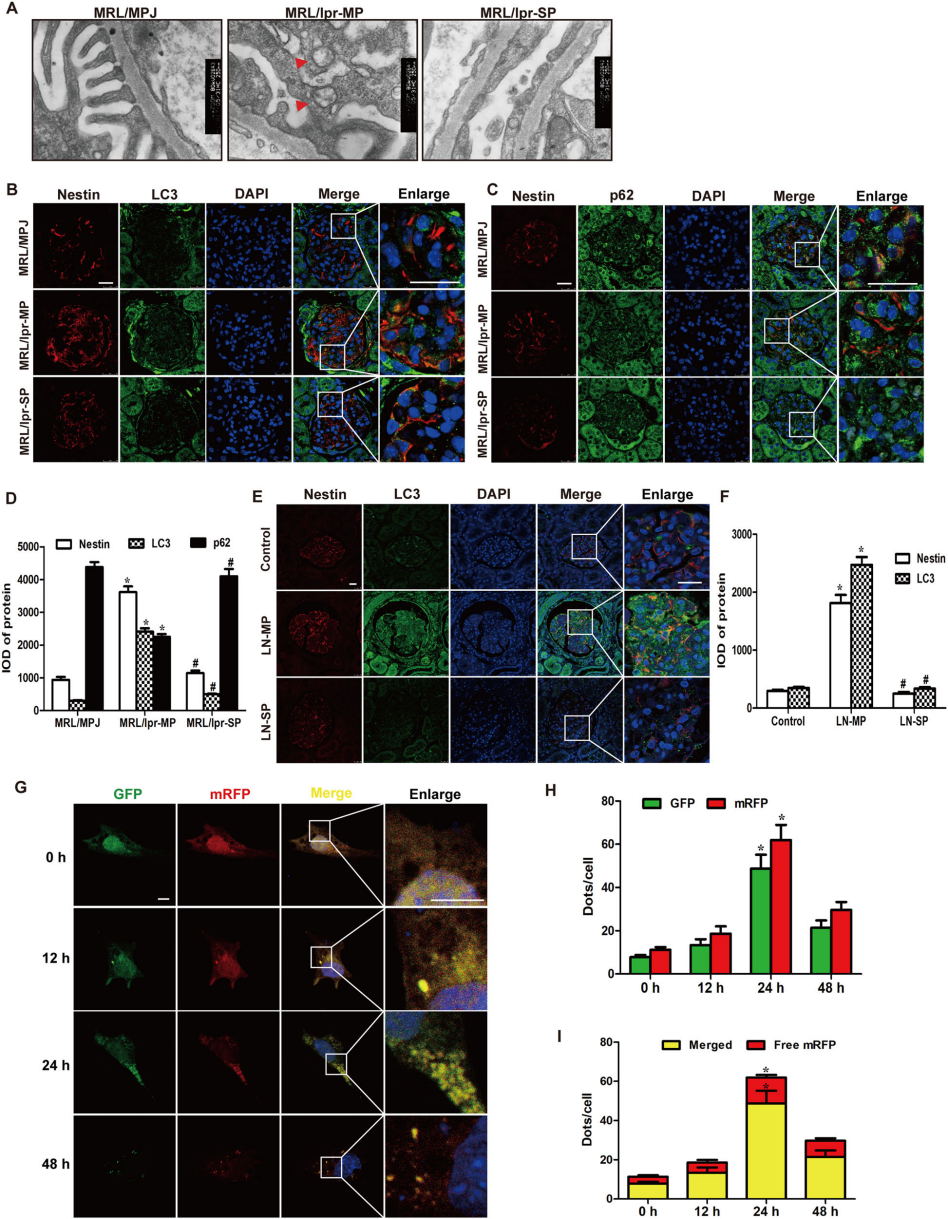
Autophagy was increased in podocytes of lupus nephritis with mild proteinuria. **a** Electron microscopy assay showed the ultrastructure of the podocytes in renal cortex of MRL/lpr mice. Red arrows referred to autophagosomes. Scale bars: 250 nm. **b**–**d** IF assay showed that the expression of nestin, LC3 and p62 in glomeruli of MRL/lpr mice. Scale bars: 25 μm. *P < 0.05 vs. MRL/MPJ mice, ^#^*P* < 0.05 vs. MRL/lpr-MP mice. **e**, **f** IF assay showed that the expression of nestin and LC3 in glomeruli of LN patients. Scale bars: 25 μm. *P < 0.05 vs. control group, ^#^*P* < 0.05 vs. LN-MP group. **g** IF assay showed that the autophagosome after mRFP-GFP-LC3 plasmid transfected to MPCs stimulated with LN plasma. Scale bars: 10 μm. **h** Quantitative data for green or red puncta per cell after mRFP-GFP-LC3 plasmid transfected to MPCs stimulated with LN plasma. *P < 0.05 vs. 0 h group. **i** Quantitative data for yellow or free red puncta per cell after mRFP-GFP-LC3 plasmid transfected to MPCs stimulated with LN plasma. *P < 0.05 vs. 0 h group. Bonferroni’s correction was performed to analyze statistical significance. Values are mean ± SEM.

In histopathological examination of the kidneys in MRL/lpr mice, we found a large number of autophagosomes in the podocytes with slight foot fusion in MRL/lpr-MP mice, but few autophagosomes in the podocytes with severe foot fusion in MRL/lpr-SP mice using TEM (Fig. 3a). Studies reported that increased autophagy had a cytoprotective effect against antibodies and interferon-α-induced podocyte injury in LN^16^. Is the effect of nestin on podocyte injury related to autophagy? As shown in Fig. 3b, c, either LC3 or p62 was colocated with nestin. In the meantime, nestin and LC3 expression both increased and p62 decreased in the MRL/lpr-MP mice compared with the MRL/MPJ group (Fig. 3b–d). Nevertheless, nestin and LC3 decreased and p62 increased in the MRL/lpr-SP mice compared with the MRL/lpr-MP mice. Moreover, the results in LN patients were consistent with mice (Fig. 3e, f).

**Fig. 4:**
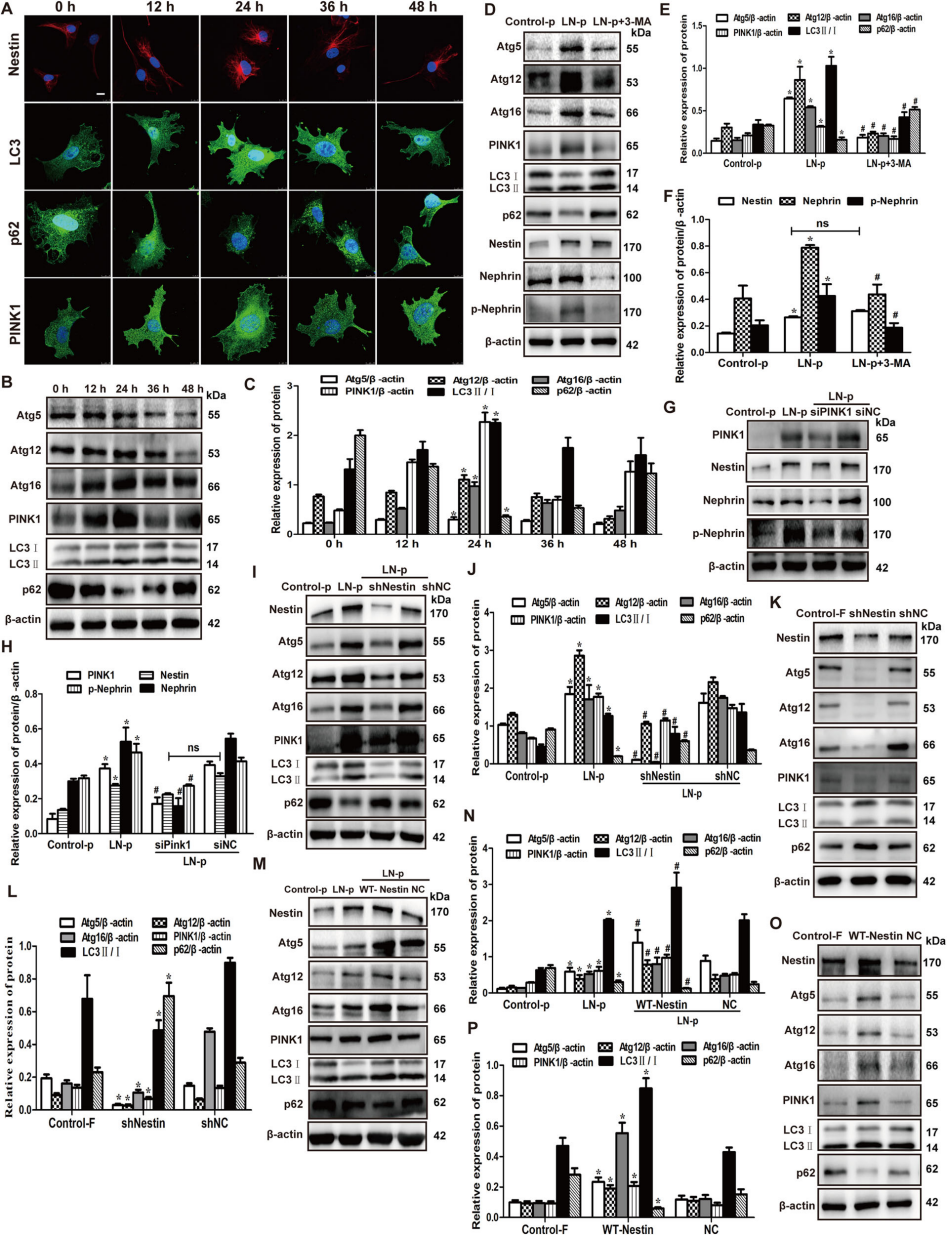
Nestin induced the mitophagy increased in lupus nephritis, and regulated the phosphorylation of nephrin by mitophagy. **a** IF assay showed that the expression of nestin, LC3, p62 and PINK1 in MPCs treated with LN plasma. Scale bars: 10 μm. **b**, **c** Western blot assay showed the expression of Atg5, Atg12, Atg16, PINK1, LC3 and p62 in the MPCs exposed to LN plasma. *P < 0.05 vs. 0 h group (*n* = 3). **d**–**f** Western blot assay showed the expression of Atg5, Atg12, Atg16, PINK1, LC3, p62, nestin, nephrin and p-nephrin (Y1217) in the MPCs, which were pretreated with 3-MA to inhibit the autophagy and exposed to LN plasma for 24 h. *P < 0.05 vs. control plasma group, ^#^*P* < 0.05 vs. LN plasma group, n.s. no significance (*n* = 3). **g**, **h** Knockdown of the expression of PINK1 effectively by siPINK1 in MPCs exposed to LN plasma for 24 h, Western blot assay showed the expression of PINK1, nestin, nephrin and p-nephrin (Y1217). *P < 0.05 vs. control plasma group, ^#^*P* < 0.05 vs. LN plasma + siNC group, n.s. no significance (*n* = 3). **i**, **j** Western blot assay showed the downregulation of nestin affected the expression of Atg5, Atg12, Atg16, PINK1, LC3 and p62 in the MPCs exposed to LN plasma for 24 h. *P < 0.05 vs. control plasma group, ^#^*P* < 0.05 vs. LN plasma + shNC group (*n* = 3). **k**, **l** Western blot assay showed the downregulation of nestin affected the expression of Atg5, Atg12, Atg16, PINK1, LC3 and p62 in the MPCs. *P < 0.05 vs. control FBS group (*n* = 3). **m**, **n** Western blot assay showed the overexpression of nestin affected the expression of Atg5, Atg12, Atg16, PINK1, LC3 and p62 in the MPCs exposed to LN plasma for 24 h. *P < 0.05 vs. control plasma group, ^#^*P* < 0.05 vs. LN plasma + NC group (*n* = 3). **o**, **p** Western blot assay showed the overexpression of nestin affected the expression of Atg5, Atg12, Atg16, PINK1, LC3 and p62 in the MPCs. *P < 0.05 vs. control FBS group (*n* = 3). Bonferroni’s correction was performed to analyze statistical significance. Values are mean ± SEM.

Moreover, we utilized the mRFP-GFP-LC3 plasmid to further confirm autophagy induction by forming puncta in vitro, which represented autophagosome formation as described^17^. As shown in Fig. 3g–i, after transfection with mRFP-GFP-LC3 plasmid, we observed the successful introduction of this plasmid showing both fluorescent proteins. In addition to accumulation of LC3, there were more red puncta in LN plasma-induced MPCs at 24 h than 0 h, which further confirmed the induction of autolysosome formation, indicating that LN plasma mediated autophagy flux in podocytes. Importantly, IF in Fig. 4a showed that nestin, PTEN-induced putative kinase protein 1 (PINK1), a key initiator of mitophagy, and LC3 expression significantly increased in LN plasma-stimulated MPCs at 24 h, while p62 decreased. In addition, the results in Fig. 4b, c revealed that PINK1, LC3, Atg5, Atg12 and Atg16 expression was upregulated, whereas p62 expression decreased in MPCs exposed to LN plasma at 24 h, and the control plasma had no effect on Atg5, Atg12, Atg16, PINK1, LC3, and p62 expression compared with the control FBS group (Supplementary Fig. S1b, d).

Subsequently, in order to explore the relationship between autophagy and nestin or nephrin, 3-MA, a specific inhibitor of autophagy, was used to inhibit the autophagy. As shown in Fig. 4d–f, 3-MA significantly inhibited the Atg5, Atg12, Atg16, PINK1, and LC3 protein, and then decreased nephrin and p-nephrin (Y1217) expression induced by LN plasma in MPCs. Importantly, PINK1-specific siRNA could decrease the PINK1 expression and inhibit the mitophagy, and knockdown of PINK1 expression also downregulated nephrin and p-nephrin (Y1217) expression induced by LN plasma (Fig. 4g, h). However, nestin was not affected by 3-MA and siPINK1. The results were similar in MPCs without LN plasma treatment (Supplementary Fig. S2). Above all, the mitophagy could affect the expression and phosphorylation of nephrin in podocyte.

Is the effect of mitophagy on nephrin mediated by nestin? Then, we knocked down the nestin expression and detected the autophagy level in MPCs. Figure 4I, j shows PINK1, Atg5, Atg12, Atg16 and LC3 expression decreased and p62 increased in the LN plasma + shNestin group compared with the LN plasma + shNC group, and nestin also regulated the autophagy-related proteins in MPCs without LN plasma (Fig. 4k, l). Simultaneously, given the nestin overexpression in MPCs by WT-Nestin plasmid, the PINK1, Atg5, Atg12, Atg16 and LC3 expression increased and p62 decreased (Fig. 4m–p). Above all, we speculated that nestin regulated the expression and phosphorylation of nephrin by mediating the mitophagy in podocyte.

### Nestin regulated the mitochondrial function by the imbalance of mitochondrial division and fusion

**Fig. 5:**
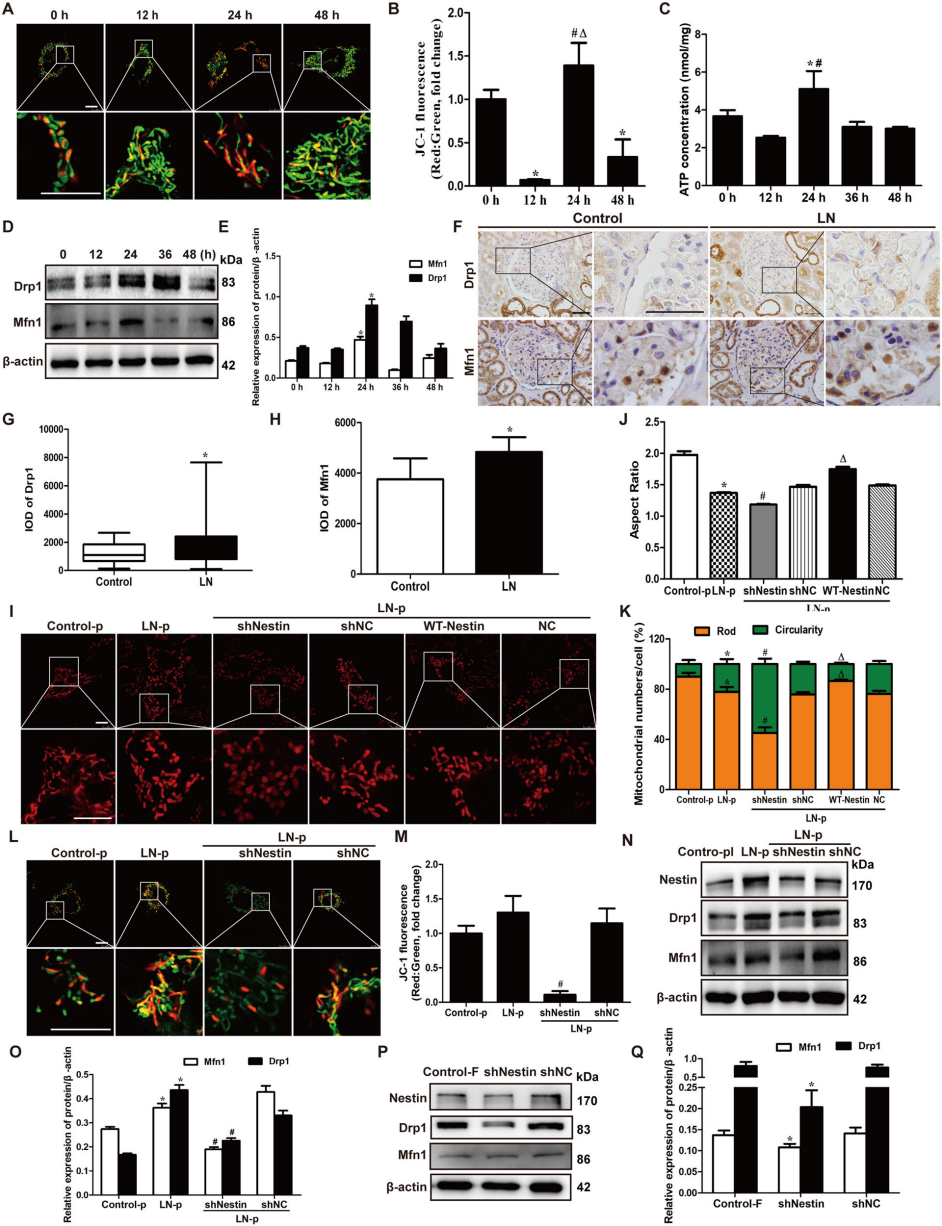
Nestin induced the imbalance of mitochondrial division and fusion, leading to mitochondrial dysfunction. **a** JC-1 staining was used to detect mitochondrial membrane potential of MPCs exposed to LN plasma. Scale bars: 10 μm. **b** Quantification of JC-1 fluorescence (red:green ratio) showing changes in fluorescence intensity in MPCs exposed to LN plasma in different time. JC-1 fluorescence was normalized with the red-to-green ratio of 0 h group. *P < 0.05 vs. 0 h group, ^#^*P* < 0.05 vs. 12 h group, ^△^*P* < 0.05 vs. 48 h group (*n* = 20). **c** ATP assay showed the ATP content in MPCs exposed to LN plasma. *P < 0.05 vs. 12 h group, ^#^*P* < 0.05 vs. 48 h group (*n* = 3). **d**, **e** Western blot assay showed the expression of Drp1 and Mfn1 in the MPCs exposed to LN plasma. *P < 0.05 vs. 0 h group (*n* = 3). **f**–**h** IHC staining showed the expression of Drp1 and Mfn1 in LN patients. Scale bars: 50 μm. *P < 0.05 vs. control group. **i** Mito Tracker staining showed the mitochondria morphology of MPCs after nestin overexpression and downregulation and exposed to LN plasma for 24 h. Scale bars: 10 μm. **j** Quantification of Mito Tracker staining (aspect ratio) in MPCs in different treatment. *P < 0.05 vs. control plasma group, ^#^*P* < 0.05 vs. LN plasma + shNC group, ^△^*P* < 0.05 vs. LN plasma + NC group (*n* = 360). **k** Quantification of Mito Tracker staining showing the different morphology mitochondrial numbers in MPCs in different treatment. *P < 0.05 vs. control plasma group, ^#^*P* < 0.05 vs. LN plasma + shNC group, ^△^*P* < 0.05 vs. LN plasma + NC group (*n* = 20). **l** JC-1 staining was used to detect mitochondrial membrane potential of MPCs after downregulation of the nestin expression and exposed to LN plasma for 24 h. Scale bars: 10 μm. **m** Quantification of JC-1 fluorescence (red:green ratio) showing changes in fluorescence intensity in MPCs in different treatment. JC-1 fluorescence was normalized with the red-to-green ratio of the control plasma group. #*P* < 0.05 vs. LN plasma + shNC group (*n* = 20). **n**, **o** Western blot assay showed the downregulation of nestin affected the expression of Drp1 and Mfn1 in the MPCs exposed to LN plasma for 24 h. *P < 0.05 vs. control plasma group, ^#^*P* < 0.05 vs. LN plasma + shNC group (*n* = 3). **p**, **q** Western blot assay showed the downregulation of nestin affected the expression of Drp1 and Mfn1 in the MPCs. *P < 0.05 vs. control FBS group (*n* = 3). Student’s *t* test and Mann−Whitney nonparametric tests were applied to compare variables between two groups. Bonferroni’s correction was performed to analyze statistical significance between multiple comparisons.

To verify the reason why nestin could regulate the mitophagy and in turn contributed to the expression and phosphorylation of nephrin, mitochondrial structure and function were detected. Interestingly, JC-1 fluorescence detection showed a decrease of the red fluorescence that was accumulated in the mitochondria and an increase of the green fluorescence distributed in the cytoplasm in MPCs exposed to LN plasma at 12 and 48 h compared to the 0 h group, which indicated that LN plasma decreased the MMP (Fig. 5a, b). However, the MMP increased in MPCs cultured with LN plasma at 24 h in contrast to 12 and 48 h, thus suggesting MMP changes in a wavy trend. Then, ATP levels significantly increased in the MPCs cultured with LN plasma at 24 h compared with 12 and 48 h (Fig. 5c). In addition, Western blot showed the expression of Mfn1 and Drp1, mitochondrial fusion and division protein, increased in MPCs exposed to LN plasma at 24 h (Fig. 5d), and compared with the control FBS group, there was no difference in Mfn1 and Drp1 expression in the control plasma group (Supplementary Fig. S1b, e). As shown in Fig. 5f–h, Mfn1 and Drp1 expression increased in glomeruli of LN patients compared to the control group. In addition, Mito Tracker Red showed that mitochondrial morphology was spherical with increased circularity and decreased aspect ratios in MPCs of the LN plasma group (Fig. 5i–k). Based on the above results, there was an imbalance of mitochondrial fusion and division in podocyte of LN, which activate the mitophagy to remove the damaged mitochondria. However, once decompensated, mitochondrial damage leads to mitochondrial dysfunction. In order to explore the role of nestin in mitochondrial injury in LN, we knocked down nestin expression in MPCs. The number of spherical mitochondria (Fig. 5i–k) was increased in the LN plasma + shNestin group compared with the LN plasma + shNC group, whereas the MMP decreased (Fig. 5l, m), and Mfn1 and Drp1 expression decreased (Fig. 5n–q). In contrast, more mitochondria became rod-shaped in MPCs stimulated with LN plasma and transfected by WT-Nestin plasmid (Fig. 5i–k).

### Mitophagy and ROS production induced by LN plasma interacted with each other

**Fig. 6:**
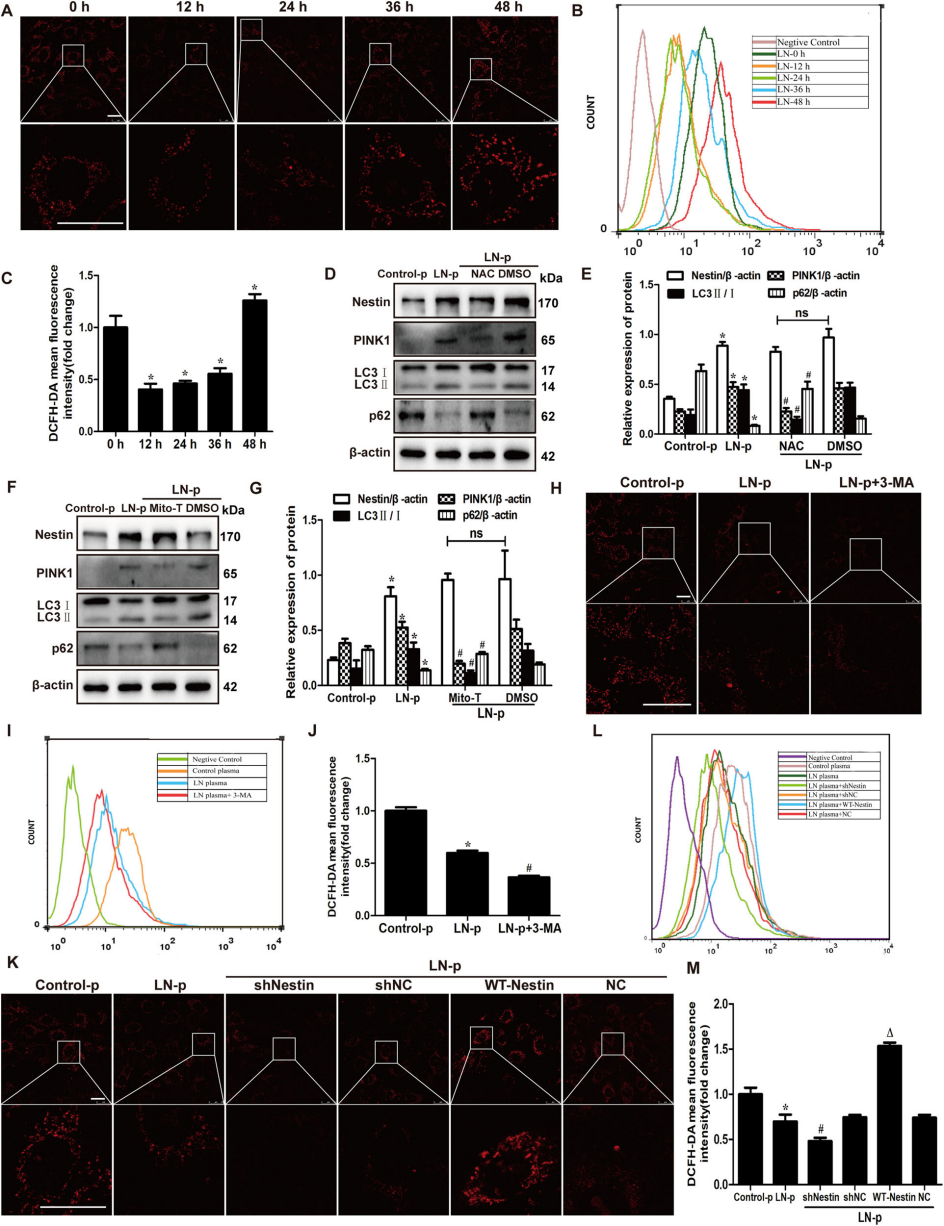
Nestin could regulate interacted ROS production and mitophagy caused by LN plasma. **a**, **b** MitoSOX and FCM assay showed the ROS production of MPCs exposed to LN plasma. Scale bars: 25 μm. **c** Quantification of DCFH-DA mean fluorescence intensity showing changes in fluorescence intensity in MPCs exposed to LN plasma in different times. DCFH-DA fluorescence was normalized with the mean fluorescence intensity of 0 h group. *P < 0.05 vs. 0 h group (*n* = 3). **d**, **e** Western blot assay showed the expression of nestin, PINK1, LC3 and p62 in the MPCs, which were pretreated with NAC and exposed to LN plasma for 24 h. *P < 0.05 vs. control plasma group, ^#^*P* < 0.05 vs. LN plasma + DMSO group, n.s. no significance (*n* = 3). **f**, **g** Western blot assay showed the expression of nestin, PINK1, LC3 and p62 in the MPCs, which were pretreated with Mito-TEMPO and exposed to LN plasma for 24 h. *P < 0.05 vs. control plasma group, ^#^*P* < 0.05 vs. LN plasma + DMSO group, n.s. no significance (*n* = 3). **h**, **i** MitoSOX and FCM assay showed the ROS production of MPCs, which were pretreated with 3-MA and exposed to LN plasma for 24 h. Scale bars: 25 μm. **j** Quantification of DCFH-DA mean fluorescence intensity showing changes in fluorescence intensity in MPCs exposed to different treatment. DCFH-DA fluorescence was normalized with the mean fluorescence intensity of control plasma group. *P < 0.05 vs. control plasma group, ^#^*P* < 0.05 vs. LN plasma group (*n* = 3). **k**, **l** MitoSOX staining and FCM showed the upregulation and downregulation of nestin affected the production of ROS in the MPCs exposed to LN plasma for 24 h. Scale bars: 25 μm. **m** Quantification of DCFH-DA mean fluorescence intensity showing changes in fluorescence intensity in MPCs exposed to different treatments. DCFH-DA fluorescence was normalized with the mean fluorescence intensity of the control plasma group. *P < 0.05 vs. control plasma group, ^#^*P* < 0.05 vs. LN plasma + shNC group, ^△^*P* < 0.05 vs. LN plasma + NC group (*n* = 3). Bonferroni’s correction was performed to analyze statistical significance. Values are mean ± SEM.

To determine whether mitochondrial dysfunction could affect ROS generation in MPCs cultured with LN plasma, we examined cellular ROS production. MitoSOX and FCM showed that ROS production was decreased in MPCs stimulated with LN plasma at 24 h and increased at 48 h compared to the 0 h group (Fig. 6a–c). Would the ROS production affect mitophagy? Subsequently, in order to explore the relationship between ROS production and mitophagy or nestin, NAC, an antioxidant compound, and Mito-TEMPO, a mitochondria-targeted superoxide dismutase mimetic with superoxide and alkyl radical scavenging properties, were used. The PINK1 and LC3 expression decreased and the p62 increased in MPCs pretreated with NAC and Mito-TEMPO and stimulated with LN plasma (Fig. 6d–g). However, the nestin expression was not affected by NAC and Mito-TEMPO. The results were similar in MPCs without LN plasma stimulation (Supplementary Fig. S3). ROS production could affect mitophagy in MPCs, and what is the effect of mitophagy on ROS production? MitoSOX and FCM showed that ROS production was decreased in MPCs pretreated with 3-MA (Fig. 6h–j), which indicated that mitophagy could affect the ROS production. Importantly, the ROS generation was much lower once knocking down the nestin expression, and increased due to overexpression of nestin (Fig. 6k–m). Taken together, these results indicated that mitophagy and ROS production could interact with each other, and nestin could regulate the mitophagy and ROS production.

### Abnormal ROS production by mitochondria broke the PTP1B/Fyn balance and promoted phosphorylation of nephrin

**Fig. 7:**
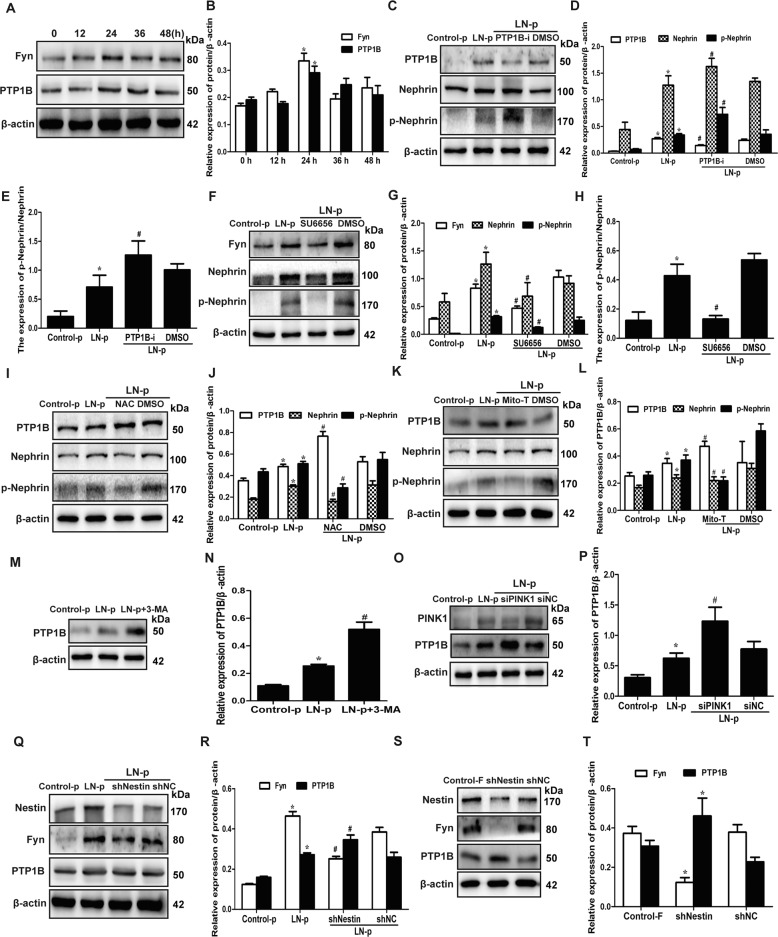
Abnormal ROS production induced the PTP1B/Fyn imbalance and promoted phosphorylation of nephrin. **a**, **b** Western blot assay showed the expression of PTP1B and fyn in the MPCs exposed to LN plasma. *P < 0.05 vs. 0 h group (*n* = 3). **c**–**e** Western blot assay showed the expression of PTP1B, nephrin and p-nephrin (Y1217) in the MPCs, which were pretreated with PTP1B inhibitor to inhibit the expression of PTP1B and exposed to LN plasma for 24 h. *P < 0.05 vs. control plasma group, ^#^*P* < 0.05 vs. LN plasma + DMSO group (*n* = 3). **f**–**h** Western blot assay showed the expression of fyn, nephrin and p-nephrin (Y1217) in the MPCs, which were pretreated with SU6656 to inhibit the expression of fyn and exposed to LN plasma for 24 h. *P < 0.05 vs. control plasma group, ^#^*P* < 0.05 vs. LN plasma + DMSO group (*n* = 3). **i**, **j** Western blot assay showed the expression of PTP1B, nephrin and p-nephrin (Y1217) in the MPCs, which were pretreated with NAC and exposed to LN plasma for 24 h. *P < 0.05 vs. control plasma group, ^#^*P* < 0.05 vs. LN plasma + DMSO group (*n* = 3). **k**, **l** Western blot assay showed the expression of PTP1B, nephrin and p-nephrin (Y1217) in the MPCs, which were pretreated with Mito-TEMPO and exposed to LN plasma for 24 h. *P < 0.05 vs. control plasma group, ^#^*P* < 0.05 vs. LN plasma + DMSO group (*n* = 3). **m**, **n** Western blot assay showed the expression of PTP1B in the MPCs, which were pretreated with 3-MA to inhibit the autophagy and exposed to LN plasma for 24 h. *P < 0.05 vs. control plasma group, ^#^*P* < 0.05 vs. LN plasma group (*n* = 3). **o**, **p** Knockdown of the expression of PINK1 effectively by siPINK1 in MPCs exposed to LN plasma for 24 h, Western blot assay showed the expression of PTP1B. *P < 0.05 vs. control plasma group, ^#^*P* < 0.05 vs. LN plasma + siNC group (*n* = 3). **q**, **r** Western blot assay showed the downregulation of nestin affected the expression of PTP1B and fyn in the MPCs exposed to LN plasma for 24 h. *P < 0.05 vs. control plasma group, ^#^*P* < 0.05 vs. LN plasma + shNC group (*n* = 3). **s**, **t** Western blot assay showed the downregulation of nestin affected the expression of PTP1B and fyn in the MPCs. *P < 0.05 vs. control FBS group (*n* = 3). Bonferroni’s correction was performed to analyze statistical significance. Values are mean ± SEM.

Phosphorylation and dephosphorylation of nephrin tyrosine residues are mainly regulated by the balance of fyn and PTP1B^13^. PTP1B, a member of the PTPs family, is a group of ROS-sensitive enzymes. PTPs lose activity when endogenous ROS increases, while PTPs restore phosphatase activity when endogenous ROS is reduced. The fyn and PTP1B expression increased in MPCs stimulated with LN plasma at 24 h compared with the 0 h group (Fig. 7a, b), whereas the ROS production decreased (Fig. 6a–c). The PTP1B expression was opposite to ROS production in MPCs. Then, the MPCs were pretreated with PTP1B inhibitor and SU6656, a selective Src family kinase inhibitor, followed by stimulation with LN plasma to reveal the effect of PTP1B and fyn on the nephrin phosphorylation. Western blot showed PTP1B inhibitor promoted nephrin phosphorylation, which, however, was abolished by SU6656 in MPCs (Fig. 7c–h). In the meantime, the nephrin expression was also affected by PTP1B and fyn, and was consistent with the change trend of p-nephrin, and the ratio of p-nephrin to total nephrin increased in the LN plasma + PTP1B inhibitor group and decreased in the LN plasma + SU6656 group compared with the LN plasma + DMSO group. The results were similar in MPCs without LN plasma treatment (Supplementary Fig. S4), and compared with the control FBS group, there was no difference in the PTP1B and fyn expression in the control plasma group (Supplementary Fig. S1b, e).

Subsequently, the PTP1B expression increased, and nephrin and p-nephrin (Y1217) decreased in the MPCs pretreated with NAC and Mito-TEMPO and stimulated by LN plasma (Fig. 7i–l), and the results were similar in MPCs without LN plasma stimulation (Supplementary Fig. S3). Simultaneously, the PTP1B expression increased in the LN plasma + 3-MA and the LN plasma + siPINK1 group (Fig. 7m–p), and the ROS generation was much lower once inhibiting the autophagy (Fig. 6h–j). And the PTP1B expression increased in the 3-MA and siPINK1 group without LN plasma stimulation (Supplementary Fig. S2). Above all, these results indicated that ROS production affected PTP1B activity, thereby inducing the imbalance of PTP1B/Fyn, and thus participating in the occurrence of tyrosine phosphorylation of nephrin.

In order to explore the role of nestin in ROS-induced PTP1B/Fyn imbalance, we knocked down the nestin expression in MPCs, as the ROS generation was much lower once knocking down the nestin expression (Fig. 6k–m). The fyn expression was reversed and PTP1B further increased in MPCs, which knocked down the nestin expression (Fig. 7q–t). Together, these results suggested that nestin-regulated mitophagy might protect mitochondrial function, whereas ROS produced by the imbalance of mitochondrial function broke the PTP1B/Fyn balance and subsequently promoted phosphorylation of nephrin.

### Nestin protected podocytes against injury and ameliorated proteinuria in MRL/lpr mice

**Fig. 8:**
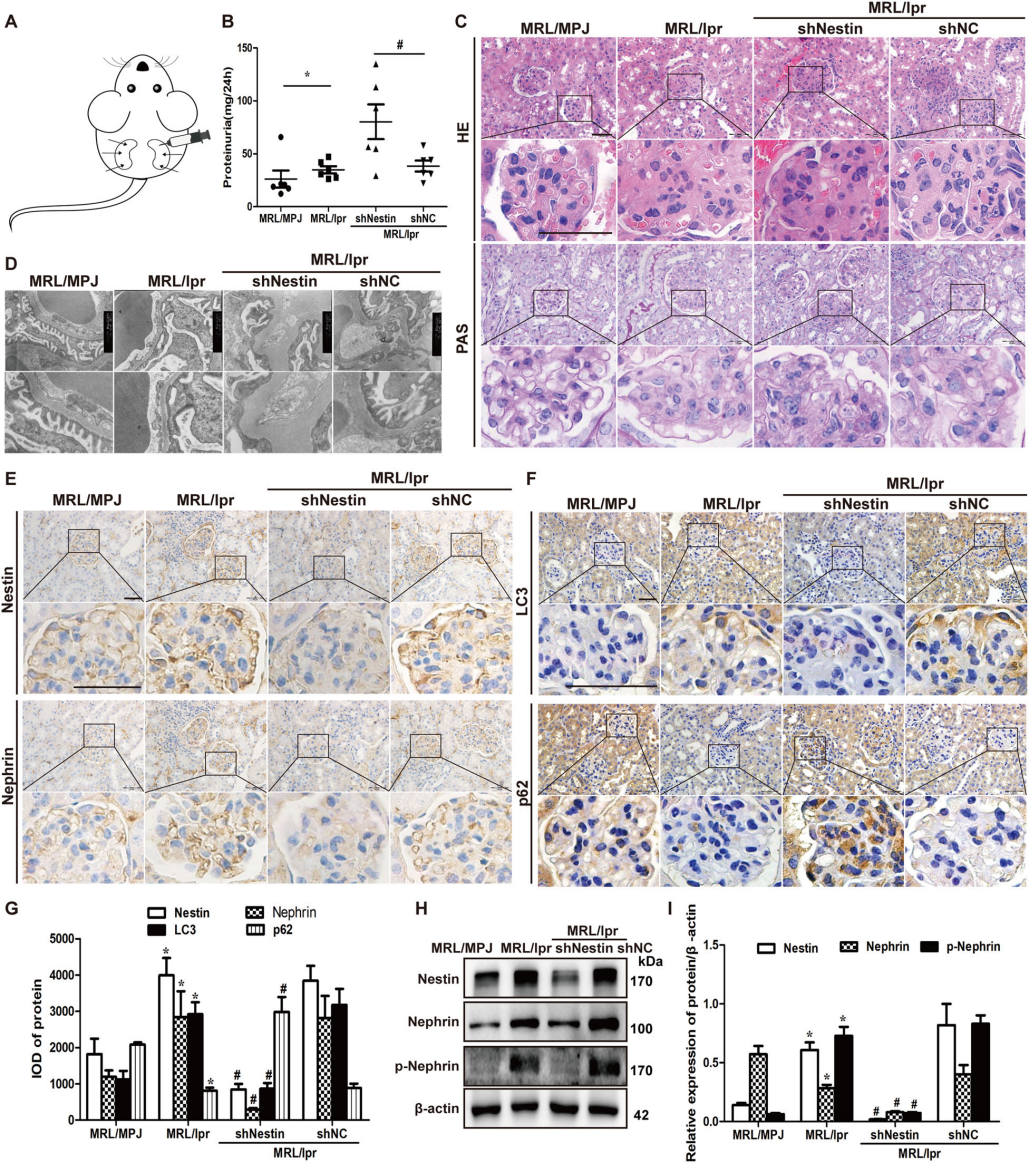
Nestin promoted the expression and phosphorylation of nephrin to ameliorate proteinuria in MRL/lpr mice. **a** Eighteen 27-week-old mice were randomly divided into three groups: MRL/lpr group (*n* = 6); MRL/lpr + shNestin group (*n* = 6); MRL/lpr + shNC group (*n* = 6). The mice in the MRL/lpr + shNestin group and the MRL/lpr + shNC group were renally injected with 50 μL 1 × 10^9^ infective units of adenoviruses in both kidneys at three sites, six MRL/MPJ mice enrolled as control mice, and six MRL/lpr mice were injected with isometric saline. Three weeks later, the mice were sacrificed. **b** Linear regression showed the 24 h proteinuria of MRL/MPJ and MRL/lpr mice by ELISA method. *P < 0.05 vs. MRL/MPJ mice, ^#^*P* < 0.05 vs. MRL/lpr + shNC mice. Every group *n* = 6. **c** HE and PAS staining showed the kidney histopathology of the MRL/lpr mice. Scale bars: 50 μm. **d** Electron microscopy assay showed the ultrastructure of the podocytes in renal cortex of the MRL/lpr mice. Scale bars: 667 nm. **e**–**g** IHC staining showed the expression of nestin, nephrin, LC3 and p62 in the MRL/lpr mice. Scale bars: 50 μm. *P < 0.05 vs. MRL/MPJ group, ^#^*P* < 0.05 vs. MRL/lpr + shNC group. **h**, **i** Western blot assay showed the downregulation of nestin affected the expression of nestin, nephrin, and p-nephrin (Y1217) in MRL/lpr mice. *P < 0.05 vs. MRL/MPJ group, ^#^*P* < 0.05 vs. MRL/lpr + shNC group (*n* = 3). Bonferroni’s correction was performed to analyze statistical significance. Values are mean ± SEM.

To further explore the protection of nestin against podocytes injury by promoting the expression and phosphorylation of nephrin in vivo, MRL/lpr mice with mild proteinuria was renally injected with Nestin-shRNA-Ad (Fig. 8a). We found that knockdown of nestin expression in podocytes could aggravate proteinuria in the MRL/lpr mice (Fig. 8b). HE and PAS staining showed larger renal glomeruli volume, mesangial cell expansion, and matrix accumulation in the MRL/lpr mice. However, the pathological changes were strengthened by the knockdown of nestin (Fig. 8c). TEM revealed that knockdown of nestin aggravated the lesions and destroyed the integrity of podocytes by aggravating the foot process fusion (Fig. 8d). In addition, the nestin, nephrin and LC3 expression was increased in the MRL/lpr mice compared to the MRL/MPJ mice, and the p62 decreased (Fig. 8e–g). However, the results were reversed after the knockdown of nestin. Meanwhile, the p-nephrin expression was increased in the MRL/lpr mice, and decreased after the knockdown of nestin (Fig. 8h, i). Taken together, these results showed that nestin protected podocytes against injury by inducing mitophagy and upregulating the expression and phosphorylation of nephrin in LN.

## Discussion

Proteinuria is the main clinical manifestation of LN and an important clinical indicator affecting the renal function of patients, and is also one of the reference indicators for judging disease activity^18,19^. Podocytes are highly specific in glomerular innate cells and play a role in glomerular basement membrane filtration barrier pore size and charge^20^. A growing body of evidence has shown that podocyte injury could cause proteinuria formation in kidney disease^21^, and podocyte injury is a frequent pathological phenomenon in many cases of proteinuria nephropathy^22^. Other researchers and our team have confirmed that podocyte injury could cause proteinuria in LN^11,23,24^. And the normal expression of marker protein of podocyte cell is a hallmark of cellular structure and functional integrity^25^.

Nestin belongs to the class VI intermediate filament cytoskeletal protein, which can be expressed in endothelial cells and renal tubular epithelial cells during kidney development. When the kidney is mature, nestin is expressed merely in differentiated and mature podocytes and maintains the morphology of podocytes^8^. Studies have shown that nestin expression in podocytes is closely related to the proteinuria in kidney diseases such as IgA nephropathy, membranous nephropathy (MN) and focal stage glomerulosclerosis^9,26^. Elsherbiny et al.^27^ have confirmed that nestin expression was upregulated at 8 weeks of type I diabetes mellitus. In contrast, Takagi et al.^28^ reported that nestin expression in podocytes was already decreased at the mild proteinuric stage of rat PAN nephrosis on day 3. At the heavy proteinuric stage on day 5, the prominent reduction of nestin was clearly determined. Our previous study about diabetic nephropathy has shown that nestin transiently increased in glomeruli of diabetic rats accompanied with less proteinuria, but nestin expression decreased and proteinuria was aggravated as the disease progressed. While high glucose (HG) stimulated podocytes, similar results were obtained, and decrease of nestin expression contributed to the apoptosis of podocytes induced by HG^11^. In this study, we explored the relationship between nestin and proteinuria in LN, and found that nestin was negatively correlated with proteinuria and positively correlated with nephrin. It is acknowledged that the abnormal expression of nephrin suggests damage to the membrane. Researches have indicated that nephrin expression decreased in glomeruli in hypertensive nephropathy, diabetic nephropathy and gestational nephropathy, and nephrin was detected in the urine of some patients with glomerular proteinuria^12^. In this study, the results revealed that nephrin expression was in line with nestin and nestin was involved in the formation of proteinuria in LN possibly through the mediation of nephrin expression.

Phosphorylated nephrin-dependent skeletal protein regulatory systems are important pathways for maintaining the homeostasis of the podocyte cytoskeletal system. Studies have found that mutation of nephrin-encoding gene *Nphs1* led to severe proteinuria and the disappearance of podocyte foot processes, which further confirmed the importance of phosphorylation of nephrin in the stability of podocyte cytoskeleton^27^. Forbes et al.^29^ found that the level of tyrosine phosphorylation of nephrin increased first and then decreased in the formation of glomerular-derived proteinuria. In this study, we also found p-nephrin (Y1217) expression was consistent with nestin in the MRL/lpr mice and podocytes exposed to LN plasma in vitro. Furthermore, regulating the phosphorylation of nephrin might be one of the mechanism by which nestin affected proteinuria of LN.

Studies have reported that increased autophagy has a cytoprotective effect against antibodies and interferon-α-induced podocyte injury in LN^16^. However, is the expression and phosphorylation of nephrin in LN related to autophagy? Autophagy is an evolutionarily conserved and genetically regulated process that regulates cell death and survival and plays an important role in physiological and pathological conditions. Almost all cells have basal levels of autophagy. In the environment of nutrient deficiency, hypoxia or pathogen invasion, autophagy activity is enhanced. The amino acids produced by degradation can be reused to maintain cell homeostasis^30^. However, abnormal autophagy could mediate disease. High-glucose stimulation could inhibit the autophagy activity of podocytes, and intraperitoneal injection of rapamycin in diabetic mice could promote podocyte autophagy and alleviate podocyte injury^31^. Studies also have reported that LN patients have significantly greater podocyte injury and discrepant autophagy levels^16,32^. One of the novel findings in the present study was that a large number of bilayer membrane autophagosomes were observed in the podocytes of MRL/lpr mice with low proteinuria. In addition, the level of autophagy in the MRL/lpr mice with low proteinuria was upregulated, which was consistent with changes of nestin and nephrin expression. In vitro, inhibition of autophagy or mitophagy could reverse the nephrin expression and phosphorylation, but had no effect on nestin. Our experimental results confirmed that nestin affected the expression and phosphorylation of nephrin through mitophagy in podocyte of LN.

Phosphorylation and dephosphorylation of nephrin tyrosine residues are primarily regulated by fyn and PTP1B, and the latter is sensitive to ROS. Mitochondria act as an energy center and mitochondrial dysfunction directly leads to cytopathic energy metabolism disorders. In addition, mitochondria produce a vast majority of ROS and become important signal transduction regulators. When mitochondria were damaged, ROS production was increased significantly. However, a severe division or fusion disorder of mitochondria contributed to blocking the oxidative phosphorylation, decreasing ATP production and reducing mitochondrial-derived ROS^33^. Li et al.^34^ revealed that impaired mitochondrial function of podocytes and abnormal ROS production in diabetes led to activation of the mitophagy to remove the damaged mitochondria. In this study, the imbalance of mitochondrial division and fusion in podocyte of LN and mitochondrial dysfunction reduced ROS production. Simultaneously, the PTP1B and fyn expression was upregulated followed with an increase of p-nephrin. Additionally, nestin mediated mitochondrial fusion and division in podocytes of LN and caused mitochondrial dysfunction. Furthermore, knockdown of nestin aggravated the mitochondrial dysfunction and reduced the Drp1 and Mfn1 expression. As known, mitophagy is an important form to clear damaged mitochondria and maintain intracellular stability^35,36^, which is triggered by PINK1. Our results suggested that imbalance of mitochondrial division and fusion caused the dysfunction of mitochondria, thereby inducing the mitophagy and abnormal ROS production, and then breaking the PTP1B/Fyn balance. At the same time, mitophagy and ROS production could affect each other, and regulated the expression and phosphorylation of nephrin in podocyte.

In conclusion, our findings demonstrated that nestin played a crucial role in podocyte-dependent proteinuria by regulating the expression and phosphorylation of nephrin in LN. Mitophagy and oxidative stress may be one of the mechanisms (Supplementary Fig. S5). Nestin is expected to be a therapeutic target for relieving proteinuria of LN.

## Supplementary information

Supplementary Figure Legends

Figure S1

Figure S2

Figure S3

Figure S4

Figure S5

## References

[1] Doria A, Gatto M, Zen M, Iaccarino L, Punzi L (2014). Optimizing outcome in SLE: treating-to-target and definition of treatment goals. Autoimmun. Rev..

[2] Hajji M (2017). Factors associated with relapse of lupus nephritis: a single center study of 249 cases. Saudi J. Kidney Dis. Transpl..

[3] Ayoub I, Birmingham D, Rovin B, Hebert L (2019). Commentary on the current guidelines for the diagnosis of lupus nephritis flare. Curr. Rheumatol. Rep..

[4] Nagase M (2006). Enhanced aldosterone signaling in the early nephropathy of rats with metabolic syndrome: possible contribution of fat-derived factors. J. Am. Soc. Nephrol..

[5] Matsui I (2009). Active vitamin D and its analogue, 22-oxacalcitriol, ameliorate puromycin aminonucleoside-induced nephrosis in rats. Nephrol. Dial. Transpl..

[6] Tomioka M (2010). Nestin is a novel marker for renal tubulointerstitial injury in immunoglobulin a nephropathy. Nephrology.

[7] Filipovic N (2017). Immunohistochemical and electronmicroscopic features of mesenchymal-to-epithelial transition in human developing, postnatal and nephrotic podocytes. Histochem. Cell Biol..

[8] Chen J (2006). Differential expression of the intermediate filament protein nestin during renal development and its localization in adult podocytes. J. Am. Soc. Nephrol..

[9] Sun Y (2014). The expression and significance of neuronal iconic proteins in podocytes. PLoS ONE.

[10] Eladl MA, Elsaed M, Atef W, El-Sherbiny H, Ultrastructural M (2017). changes and nestin expression accompanying compensatory renal growth after unilateral nephrectomy in adult rats. Int. J. Nephrol. Renovasc. Dis..

[11] Liu W (2012). Nestin protects mouse podocytes against high glucose-induced apoptosis by a cdk5-dependent mechanism. J. Cell Biochem..

[12] Hauser PV, Collino F, Bussolati B, Camussi G (2009). Nephrin and endothelial injury. Curr. Opin. Nephrol. Hypertens..

[13] Aoudjit L (2011). Podocyte protein, nephrin, is a substrate of protein tyrosine phosphatase 1b. J. Signal Transduct..

[14] Xiong B, Li M, Xiang S, Han L (2018). A1AR-mediated renal protection against ischemia/reperfusion injury is dependent on HSP27 induction. Int. Urol. Nephrol..

[15] Liu J (2019). Knockdown of TRIM27 expression suppresses the dysfunction of mesangial cells in lupus nephritis by FoxO1 pathway. J. Cell Physiol..

[16] Qi Y (2018). Increased autophagy is cytoprotective against podocyte injury induced by antibody and interferon-α in lupus nephritis. Ann. Rheum. Dis..

[17] Yuan K (2012). Autophagy plays an essential role in the clearance of pseudomonas aeruginosa by alveolar macrophages. J. Cell Sci..

[18] Koo HS, Kim S, Chin HJ (2016). Remission of proteinuria indicates good prognosis in patients with diffuse proliferative lupus nephritis. Lupus.

[19] Touma Z, Urowitz MB, Ibañez D, Gladman DD (2014). Time to recovery from proteinuria in patients with lupus nephritis receiving standard treatment. J. Rheumatol..

[20] Shankland SJ (2006). The podocyte’s response to injury: role in proteinuria and glomerulosclerosis. Kidney Int..

[21] Zou J (2006). Upregulation of nestin, vimentin, and desmin in rat podocytes in response to injury. Virchows Arch..

[22] Mathieson PW (2011). The podocyte as a target for therapies—new and old. Nat. Rev. Nephrol..

[23] Fu R (2017). Podocyte activation of NLRP3 inflammasomes contributes to the development of proteinuria in lupus nephritis. Arthritis Rheumatol..

[24] Sun L (2019). A20 overexpression exerts protective effects on podocyte injury in lupus nephritis by downregulating UCH-L1. J. Cell Physiol..

[25] Endlich K, Kriz W, Witzgall R (2001). Update in podocyte biology. Curr. Opin. Nephrol. Hypertens..

[26] Su W (2007). Expression of nestin in the podocytes of normal and diseased human kidneys. Am. J. Physiol. Regul. Integr. Comp. Physiol..

[27] Elsherbiny NM, El-Sherbiny M, Said E (2015). Amelioration of experimentally induced diabetic nephropathy and renal damage by nilotinib. J. Physiol. Biochem..

[28] Takagi H (2017). USP40 gene knockdown disrupts glomerular permeability in zebrafish. Am. J. Physiol. Ren. Physiol..

[29] Forbes JM (2002). Modulation of nephrin in the diabetic kidney: association with systemic hypertension and increasing albuminuria. J. Hypertens..

[30] Wang S (2014). Autophagy-related gene atg5 is essential for astrocyte differentiation in the developing mouse cortex. Embo. Rep..

[31] Xiao T (2014). Rapamycin promotes podocyte autophagy and ameliorates renal injury in diabetic mice. Mol. Cell Biochem..

[32] Jin J (2018). The novel involvement of podocyte autophagic activity in the pathogenesis of lupus nephritis. Histol. Histopathol..

[33] Wang J (2016). Nestin regulates proliferation and invasion of gastrointestinal stromal tumor cells by altering mitochondrial dynamics. Oncogene.

[34] Li W (2017). FoxO1 promotes mitophagy in the podocytes of diabetic male mice via the PINK1/Parkin pathway. Endocrinology.

[35] Matic I, Strobbe D, Di GF, Campanella M (2017). Molecular biology digest of cell mitophagy. Int. Rev. Cell Mol. Biol..

[36] Al-Waili N, Al-Waili H, Al-Waili T, Salom K (2017). Natural antioxidants in the treatment and prevention of diabetic nephropathy; a potential approach that warrants clinical trials. Redox Rep..

