# Supplementary material for: Nestin protects podocyte from injury in lupus nephritis by mitophagy and oxidative stress
Source: Cell Death Dis. 2020 May 5;11(5):319. doi: 10.1038/s41419-020-2547-4 (PMC7200703; doi:10.1038/s41419-020-2547-4)
Supplement: Supplementary file 1 — Supplementary Figure Legends [file 41419_2020_2547_MOESM1_ESM.doc]

**Supplementary Figure Legends**

**Figure S1** Distal noncancerous kidney tissues pathologically confirmed as normal tissues. Compared with FBS treated MPCs, plasma from healthy donors had no effect on nestin, nephrin, p-nephrin, autophagy-related proteins, Mfn1, Drp1, PTP1B, and fyn in MPCs. **P*<0.05 vs. Control FBS group, #*P*<0.05 vs. control plasma group, ns, no significance (n=3).

**Figure S2 a-c** Inhibition of autophagy had no effect on nestin expression, decreased nephrin and p-nephrin expression, and increased PTP1B expression in MPCs. **P*<0.05 vs. control FBS group. **d, e** The results were similar in MPCs after inhibited mitophagy. **P*<0.05 vs. siNC group, ns, no significance (n=3).

**Figure S3** Inhibition of ROS and mtROS production had no effect on nestin expression, decreased PINK1, LC3, nephrin and p-nephrin expression, and increased p62, PTP1B expression in MPCs. **P*<0.05 vs. DMSO group, ns, no significance (n=3).

**Figure S4** Inhibition of PTP1B increased the expression and phosphorylation of nephrin. And Inhibition of fyn decreased the expression and phosphorylation of nephrin. **P*<0.05 vs. DMSO group (n=3).

**Figure S5** Nestin regulated the mitophagy and the ROS in podocyte in LN, which resulted in increased nephrin expression and phosphorylation, and ameliorated proteinuria. And mitophagy and ROS could interact with each other. In addition, abnormal ROS broke the imbalance of PTPB1/Fyn, and promoted phosphorylation of nephrin.
