# Supplementary figures and images for: Nestin protects podocyte from injury in lupus nephritis by mitophagy and oxidative stress
Source: Cell Death Dis. 2020 May 5;11(5):319. doi: 10.1038/s41419-020-2547-4 (PMC7200703; doi:10.1038/s41419-020-2547-4)

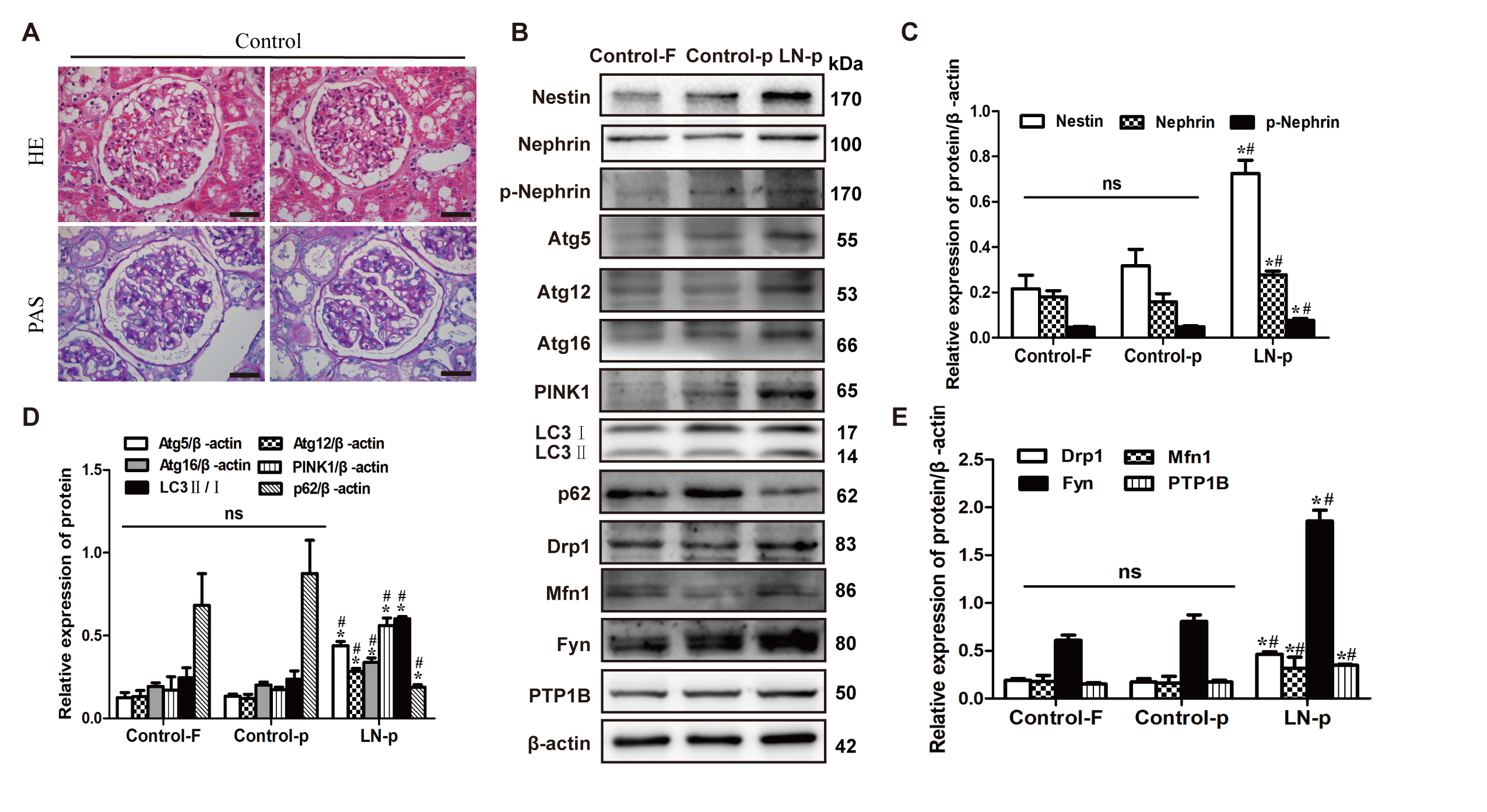

Supplement: Supplementary file 2 — Figure S1 [file 41419_2020_2547_MOESM2_ESM.png]

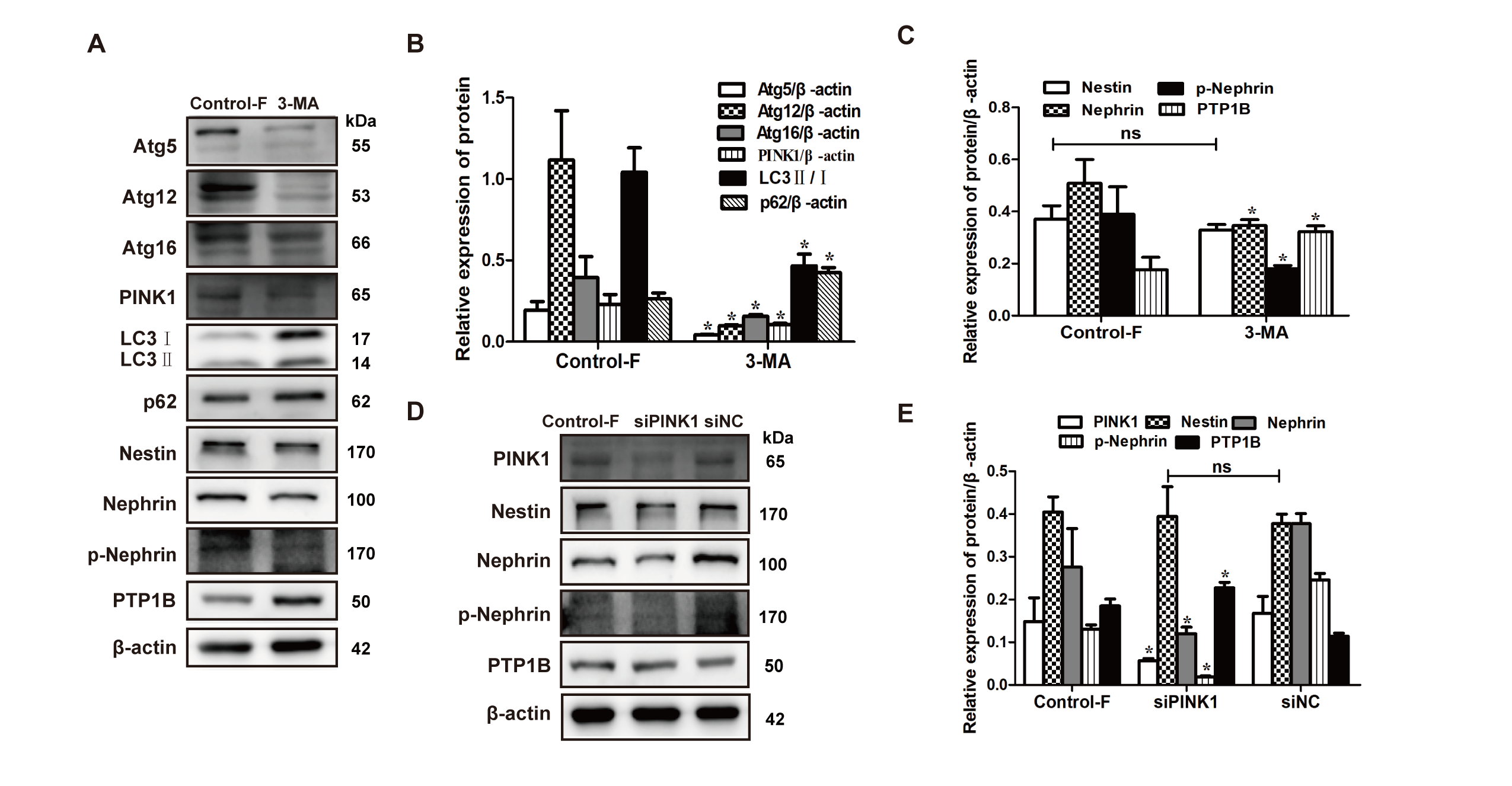

Supplement: Supplementary file 3 — Figure S2 [file 41419_2020_2547_MOESM3_ESM.png]

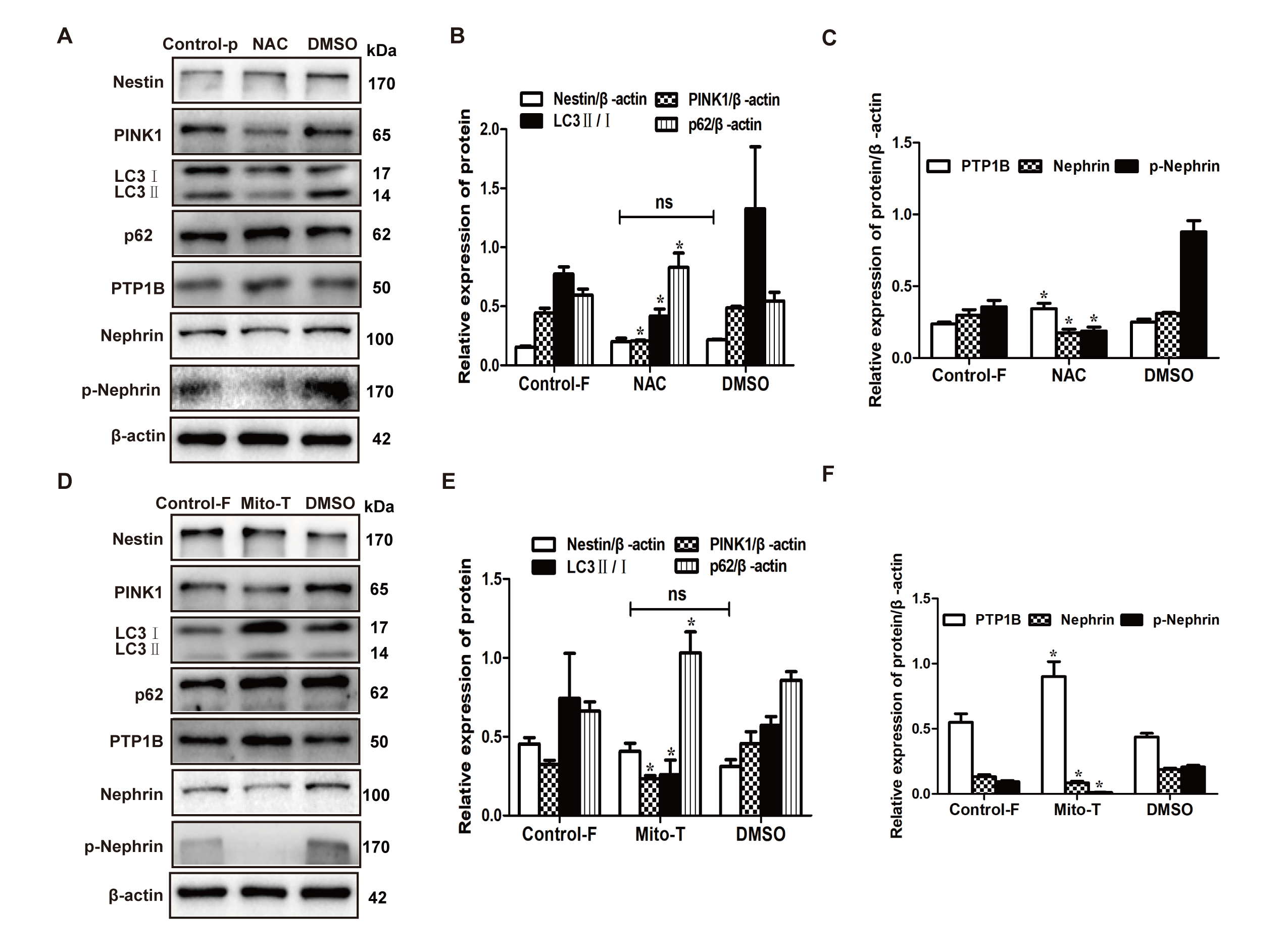

Supplement: Supplementary file 4 — Figure S3 [file 41419_2020_2547_MOESM4_ESM.png]

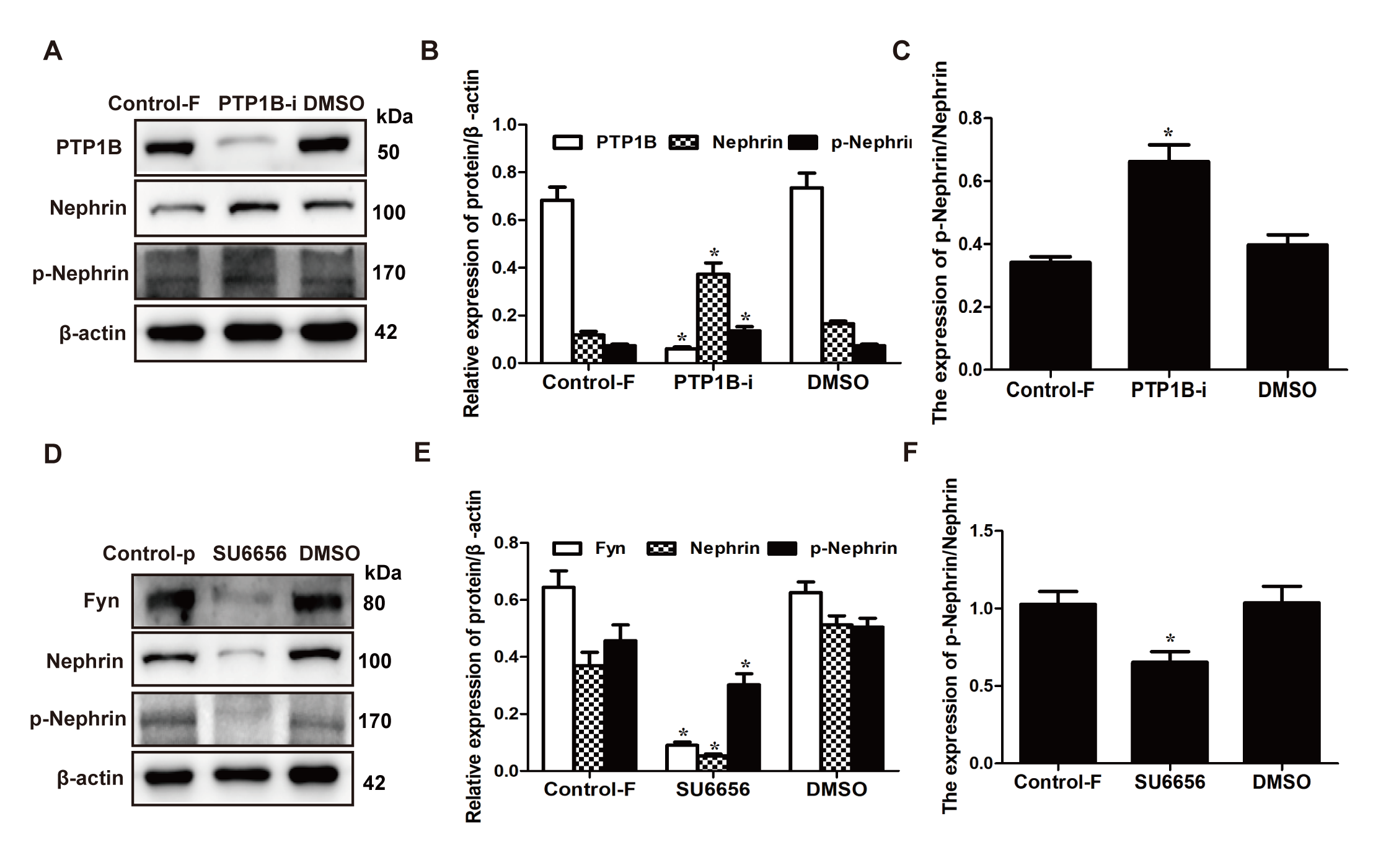

Supplement: Supplementary file 5 — Figure S4 [file 41419_2020_2547_MOESM5_ESM.png]

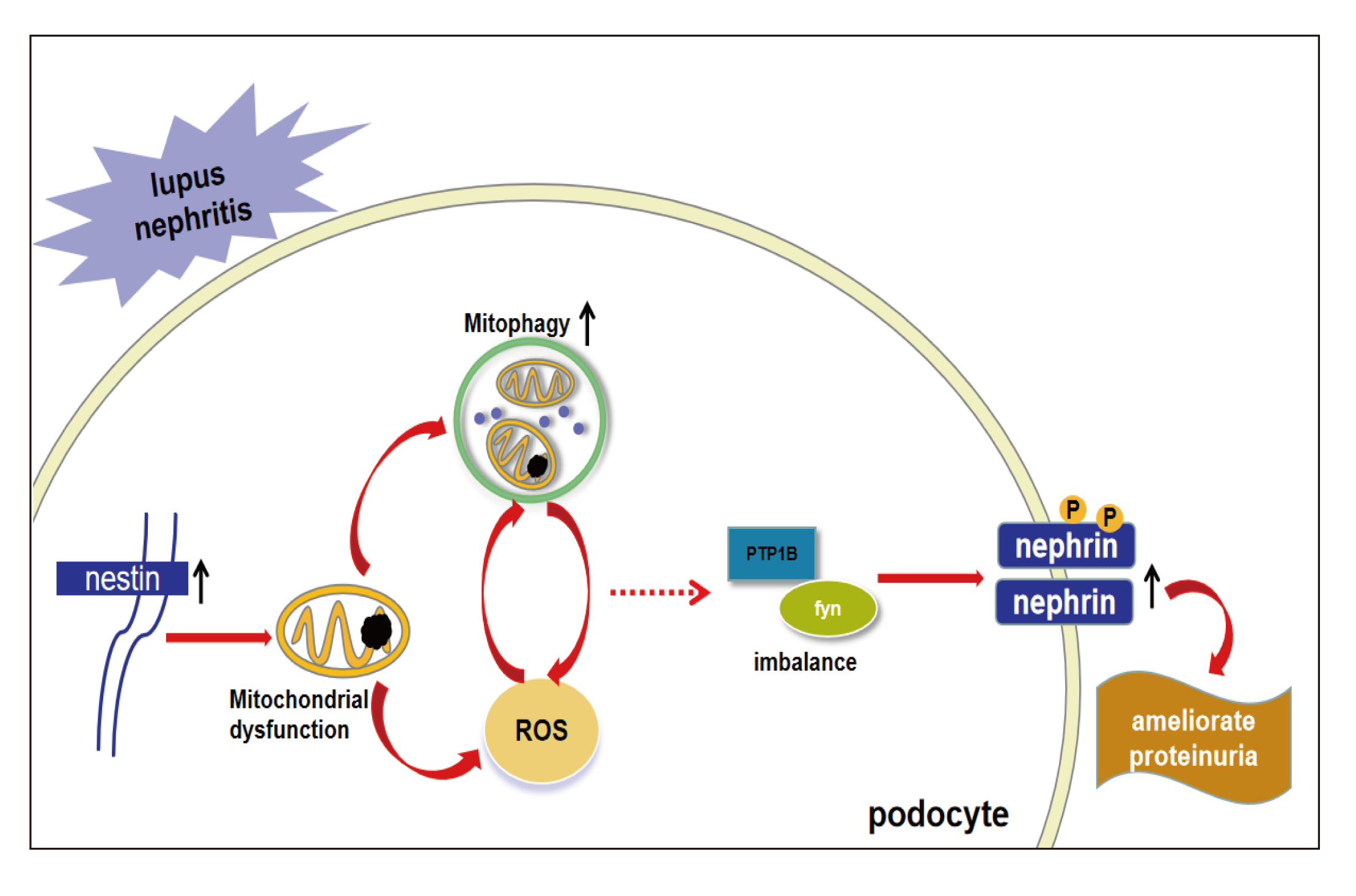

Supplement: Supplementary file 6 — Figure S5 [file 41419_2020_2547_MOESM6_ESM.png]
